# Global-best brain storm optimization algorithm based on chaotic difference step and opposition-based learning

**DOI:** 10.1038/s41598-024-56919-0

**Published:** 2024-03-18

**Authors:** Yanchi Zhao, Jianhua Cheng, Jing Cai, Bing Qi

**Affiliations:** 1 College of Intelligent Systems Science and Engineering, Harbin Engineering University, Harbin, 150001 China; 2 Beijing Institute of Space Long March Vehicle, Beijing, 100000 China

**Keywords:** Engineering, Mathematics and computing

## Abstract

Recently, the following global-best strategy and discussion mechanism have been prevailing to solve the slow convergence and the low optimization accuracy in the brain storm optimization (BSO) algorithm. However, the traditional BSO algorithm also suffers from the problem that it is easy to fall into local optimum. Therefore, this work innovatively designed the chaotic difference step strategy. This strategy introduced four commonly used chaotic maps and difference step to expand the population search space to improve the situation. Moreover, opposition-based learning thought was innovatively adopted into the BSO algorithm. The thought aims to generate the opposition-based population, increase the search density, and make the algorithm out of the local optimum as soon as possible. In summary, this work proposed a global-best brain storm optimization algorithm based on the chaotic difference step and opposition-based learning (COGBSO). According to the CEC2013 benchmark test suit, 15 typical benchmark functions were selected, and multiple sets of simulation experiments were conducted on MATLAB. The COGBSO algorithm was also compared to recent competitive algorithms based on the complete CEC2018 benchmark test suit. The results demonstrate that the COGBSO outperforms BSO and other improved algorithms in solving complex optimization problems.

## Introduction

Currently, the swarm intelligence algorithm, an emerging optimization algorithm, is indispensable to the field of artificial intelligence, which makes the population gradually move toward the optimal solution by simulating various behaviors and survival rules of the swarm^1^. Traditional swarm intelligence algorithms include but are not limited to the ant colony optimization algorithm (ACO) and the particle swarm optimization algorithm (PSO)^2,3^. With the continuous further research of swarm intelligence algorithms, such novel swarm intelligence algorithms have sprung up as the brain storm optimization algorithm, the cuckoo search algorithm (CS), the fruit fly optimization algorithm (FOA), and the firefly algorithm (FA)^4^.

**Figure 1:**
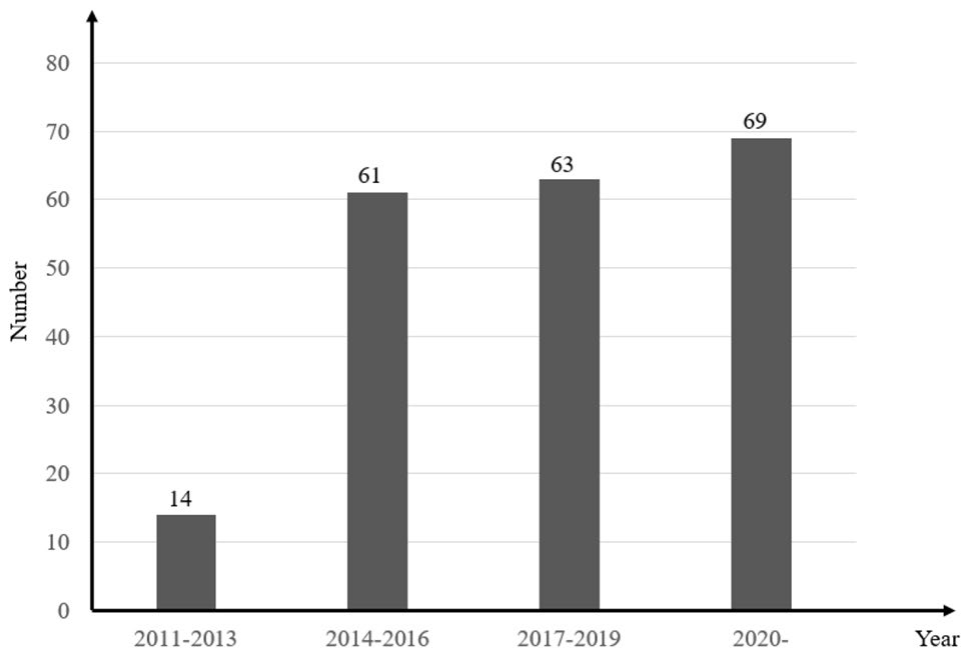
The number of papers on brain storm optimization since 2011.

Inspired by a human brainstorming conference, the BSO algorithm has received extensive academic attention regarding its high optimization accuracy and superior optimization in high dimensions^5^. The algorithm consists of four steps: clustering, substitution, selection, and mutation. Moreover, the brain storm optimization algorithm greatly improves the diversity of the population due to the use of clustering operations, which divides the population into multiple groups and makes it easier to jump out of the local optimum compared to other swarm intelligence algorithms. The BSO algorithm has succeeded in path planning^6^, image processing^7^, wireless sensor networks, and other fields^8^. To demonstrate the research value of the brain storm optimization algorithm, Fig. 1 illustrates the number of papers published every three years since its inception in 2011. As shown in this figure, only fourteen papers were published between 2011 and 2013, with a significant increase in the number of papers published between 2014 and 2016. The number of papers has been increasing recently, indicating that more and more scholars are committed to improving and applying the brain storm optimization algorithm.

Although the clustering process may enhance the population’s diversity, traditional brain storm optimization still has some disadvantages. Compared with other improved intelligence algorithms, the traditional BSO algorithm converges more slowly and fails to find the real optimal solution. A novel DE algorithm named quantum-based avian navigation optimizer algorithm (QANA) was proposed^9^. The QANA is modeled by introducing long-term and short-term memories, a V-echelon communication topology, and quantum-based navigation including two mutation strategies and a qubit-crossover operator. The experimental results show that the QANA is highly competitive with multiple intelligent algorithms. Mohammad proposed an enhanced moth-flame optimization (MFO-SFR) algorithm^10^. The algorithm introduces an effective stagnation finding and replacing (SFR) strategy to maintain population diversity effectively and improve the algorithm’s performance. Ali proposed the improved binary quantum-based avian navigation optimizer algorithm (IBQANA) based on the QANA algorithm^11^; the performance of the algorithm is further improved. A novel bio-inspired algorithm named starling murmuration optimizer (SMO) was proposed^12^. The SMO introduces a dynamic multi-flock construction and three new search strategies: separating, diving, and whirling. The SMO is applied to solve several mechanical engineering problems, and results demonstrate that it can provide more accurate solutions.

Compared to these very competitive algorithms, the performance of traditional BSO algorithms needs to be further improved. Therefore, scholars have improved the traditional BSO algorithm’s basic parameters, clustering methods, and mutation strategies. Yu presented a BSO algorithm based on an adaptive search radius to increase the convergence speed, which designed three search strategies by introducing the successful memory and failure memory to adjust the step range^13^. This algorithm is a fusion of diverse fundamental parameters designed to enhance performance for varying problems. However, its optimization accuracy increases much less than the original BSO, and this algorithm cannot solve the problem that the BSO algorithm tends to fall into the local optimum. The BSO algorithm based on elite individual guidance and parameter adaptation introduced the elite and global-best individuals to guide population mutation^14^. This algorithm sets adaptive parameters to increase the number of global mutations in the early iteration and the number of local mutations in the later iteration, whose convergence speed and optimization accuracy are significantly improved. Still, the algorithm is prone to local convergence when solving multi-modal optimization problems. El-Abd proposed the BSO algorithm based on global-best individual guidance and fitness grouping to cluster, reducing time complexity and improving performance^15^. Meanwhile, this algorithm converges slower than some of the latest improved BSO algorithms. The low convergence speed makes its optimization accuracy unsatisfactory when the iterations are limited. A BSO algorithm was proposed based on the difference-mutation and the global-best individual, where the difference step replaces the original BSO mutation step and significantly improves the convergence speed^16^. This algorithm follows the global-best mutation strategy and leads to dramatically improved optimization. However, the problem of trapping in local optimum remains, making the algorithm perform poorly in solving complex multi-modal problems. Tuba creatively introduced chaos theory and proposed a BSO algorithm based on chaotic maps to improve the BSO algorithm^17^. Compared with the original BSO algorithm, its performance slightly improves yet hardly leads to apparent advantages. However, implementing chaotic maps proposes innovative methods to address the challenge of algorithms predisposed to local convergence. Based on multi-branch chaotic maps, a BSO algorithm introduced eight chaotic maps and improved its optimization accuracy, but with low convergence speed and higher time complexity^18^. A BSO algorithm was proposed based on an adaptive self-scaling chaotic search mechanism^19^. This local search method adjusts the search space based on the adaptive self-scaling mechanism, and the chaotic local search mechanism prevents the algorithm from falling into the local optimum. Its convergence speed is improved somewhat, but the optimization accuracy is low. A BSO algorithm based on an advanced discussion mechanism was proposed^20^, which introduced a difference step strategy and simplified the selection process of the BSO algorithm. The goal of strengthening the global search in the early stage and the local search in the later stage can improve the algorithm’s optimization accuracy. Furthermore, the difference step strategy has enhanced the algorithm’s convergence speed. However, the optimization accuracy for high-dimensional multi-modal problems is short of expectation, and it is easy to fall into the local optimum trap. The global-best brain storm optimization algorithm based on discussion mechanism and difference step was proposed by reviewing the literature^21^. This algorithm combines several improvement strategies with different properties. It has optimal convergence speed and optimization accuracy compared to previous algorithms. However, it also tends to fall into local optimum when facing complex optimization problems. Therefore, the algorithm also needs further improvement.

To sum up, the improved BSO algorithms in the existing reference have such problems as low convergence speed, poor optimization accuracy, and a high probability of falling into local optimum. The low convergence speed reduces the algorithm efficiency, i.e., for a given accuracy requirement, the lower the convergence speed, the more time it will take, thus reducing the effectiveness of the practical application. Optimization accuracy is the most essential metric for testing the performance of an optimization algorithm, and low accuracy means poor algorithm performance. The algorithm may fall into the local optimum and waste a vast of time during the iteration cycle, thereby impacting the final accuracy of the optimization search. Therefore, the improvement of the brain storm optimization algorithm in this paper is to further improve the convergence speed and optimization accuracy of the algorithm based on the existing improved algorithm and to improve the ability of the algorithm to jump out of the local optimum when facing complex problems.

Overall, this work introduces opposition-based learning thought and chaos theory, fusing chaotic maps and difference steps to construct a chaotic difference step strategy. The major innovations of the paper are the algorithms’ remarkable ability to jump out of local convergence when facing complex optimization problems with multiple peaks and high dimensions and its higher convergence speed and optimization accuracy. Subsequently, a global-best BSO algorithm is proposed based on chaotic difference step and opposition-based learning. This work: (1) proposes the chaotic difference step strategy, introduces the opposition-based learning thought to generate the opposition-based population, and designs the trigger condition and end condition of the strategy to reduce the algorithm’s time complexity; (2) combines the existing global-best mutation strategy combined with the discussion mechanism to improve the convergence speed and optimization accuracy; (3) completes a large number of experiments and data analysis based on the CEC2013 and CEC2018^22,23^.

## BSO

Human brain storm conferences inspire the brain storm optimization algorithm. BSO algorithm sufficiently exerts the characteristics of human intelligence to solve problems and outperforms in convergence speed and optimization accuracy for various optimization problems. Moreover, it has more advantages than traditional optimization algorithms in high-dimensional problems. The algorithm includes four main steps: clustering, substitution, selection, and mutation.

Firstly, the K-means clustering analysis method is used. The current population of *n* solutions to enter the iteration is divided into *m* categories, and the purpose is to simulate the human group discussion behavior and improve the search efficiency of the algorithm.

Second, setting a parameter $p_{replace}$ and generating a random number $r_1$ between 0 and 1. When $r_1$ is less than $p_{replace}$, a new individual will be generated to replace the selected cluster center. If the value of $p_{replace}$ is too large, it will affect the algorithm’s convergence efficiency, reducing the population’s diversity. If this value is too small, it may cause algorithms to make the algorithm converge in advance.

Third, setting the three probability parameters $p_{one}$, $p_{one\_center}$ and $p_{two\_center}$, generating random number $r_2$,$r_3$ and $r_4$. When $r_2$ is less than $p_{one}$, select an individual in one cluster to mutate. Otherwise, select an individual from each cluster to mutate after fusion. If an individual in a cluster is selected for mutation, when $r_3$ is less than $p_{one\_center}$, the cluster center is selected for mutation; otherwise, a random individual in this cluster is selected for mutation. If individuals in two clusters are selected to mutate, when $r_4$ is less than $p_{two\_center}$, selecting the cluster centers of the two clusters to mutate; otherwise, selecting random individuals in each cluster (two individuals cannot be cluster centers at the same time) to mutate after fusion.

Fourth, fusion or mutation operations on the selected individuals are performed, and then they are compared with the original individuals according to their fitness. The outstanding individuals will be retained after the above operations. The fusion step is as follows:

1 $$\begin{aligned} {X_f} = v \times {X_1} + (1 - v) \times {X_2}, \end{aligned}$$

where ${X_f}$ is a new individual after fusion, *v* is a random number from 0 to 1, ${X_1}$ and ${X_2}$ are two random individuals to be merged. The mutation step is as follows:

2 $$\begin{aligned} {X_m} = {X_s} + \xi \times n(\mu ,\sigma ), \end{aligned}$$

where ${X_m}$ is a new individual after mutation, ${X_s}$ is the selected individual to be mutated, $n(\mu ,\sigma )$ is the Gaussian random number with the mean of $\mu$ and the variance of $\sigma$, and $\xi$ is the mutation coefficient with the mathematical expression in (3).

3 $$\begin{aligned} \xi = \log sig\left( {\frac{{0.5 \times {g_{max}} - g}}{k}} \right) \times rand(), \end{aligned}$$

where ${g_{\max }}$ is the maximum number of iterations, *g* is the current iteration number, *k* is the adjustment factor. The pseudo code of the BSO algorithm is shown in Algorithm 1.

**Algorithm 1:**
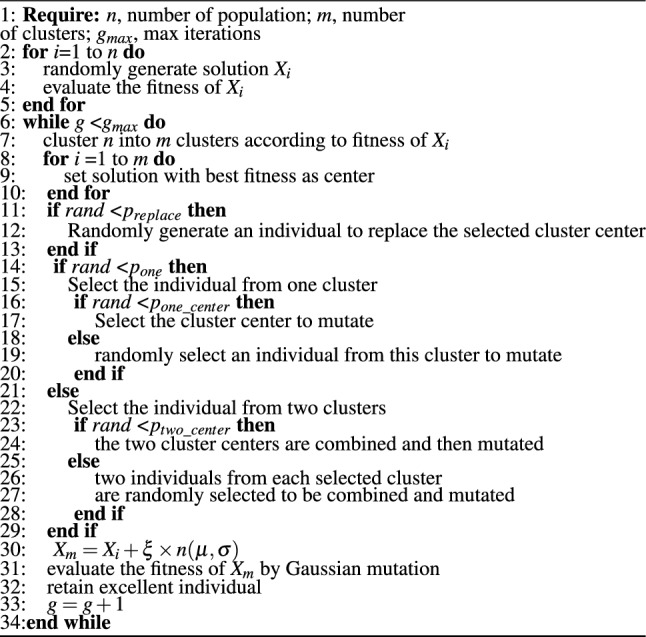
The BSO algorithm.

## COGBSO

In this section, improvements of the BSO algorithm in two aspects are introduced to improve the performance of the algorithm. The steps of the COGBSO algorithm are shown in Algorithm 2.

### Discussion mechanism based on global-best strategy

In the early iteration of intelligent algorithm search, the global search should be strengthened to improve the diversity of the population and convergence speed. The local search should be strengthened in the later iteration to improve the optimization accuracy. Because the traditional BSO algorithm’s selection process is entirely random and hard to meet the above requirements, a discussion mechanism is introduced^20^, which divides the selection process into two situations: the inter-group discussion and the intra-group discussion. An adaptive probability parameter is set to adjust the frequency of inter-group and intra-group discussion. The adaptability of the BSO algorithm is strengthened, and the algorithm’s performance is improved.

The discussion mechanism is divided into two parts: the intra-group discussion and the inter-group discussion. Intra-group discussion means that the individuals to be mutated are generated in one cluster, and it can be divided into three mutation types: random cluster center mutation, random individual mutation, and mutation after the fusion of two random individuals in the group. The inter-group discussion is that the individuals to be mutated are generated by two different clusters, including two random cluster centers fused and then mutated, two random individuals fused and then mutated, and new individual randomly generated. The new individual is randomly generated to ensure the diversity of the population and reduce the probability of the algorithm falling into the local optimum. In addition, set the adaptive probability parameter as follows:

4 $$\begin{aligned} {P_{intra}} = {P_l} + {P_r} \times (\frac{g}{{{g_{max}}}}), \end{aligned}$$

5 $$\begin{aligned} {P_{inter}} = 1 - {P_{intra}}, \end{aligned}$$

where $P_{intra}$ is the probability of intra-group discussion, $P_{inter}$ is the probability of inter-group discussion, $P_{l}$ is the lowest probability of intra-group discussion being adopted, $P_{r}$ is the linear range parameter.

The mutation step adopts the difference step. Compared with the Gaussian mutation step, the difference step has better adaptability, which can strengthen the global search ability in the early iteration and the local search ability in the later iteration. The mutation form of the difference step is as follows:

6 $$\begin{aligned} {X_m} = {X_s} + ({X_1} - {X_2}) \times rand(), \end{aligned}$$

where ${X_1}$ and ${X_2}$ are two random individuals in the population. Since there is a large gap between individuals in the early population, the difference step is large, which can improve the search range. While the later individual gap is small, a smaller difference step can make the population search in a small range and improve the optimization accuracy.

Many intelligent algorithms have applied the global-best strategy with desirable results^24,25^. The global-best strategy was introduced into the BSO algorithm for the first time^15^. The global-best individual is the individual whose fitness of each generation best meets the requirements, and the global-best strategy is to make the newly generated individual as close to the global-best individual as possible, which can improve the optimization accuracy of the algorithm. The core idea is as follows:

7 $$\begin{aligned} {X_n} = {X_m} + C \times R. \times ({X_b} - {X_m}), \end{aligned}$$

where ${X_n}$ is the new individual following the global-best individual, *R* is a vector of dimension D and each dimension is a random number from 0 to 1, ${X_b}$ is the global-best individual, *C* is the global-best coefficient that can affect the degree to which the new individual follows the global-best individual. The calculation method is as follows:

8 $$\begin{aligned} C = {C_{min}} + \frac{g}{{{g_{max}}}} \times ({C_{max}} - {C_{min}}), \end{aligned}$$,

where $C_{min}$ is the lower bound of the global-best coefficient, $C_{max}$ is the upper bound of the global-best coefficient.

### Chaotic difference step and opposition-based population strategy

The global-best strategy and discussion mechanism are combined to improve the traditional BSO algorithm, which can significantly improve the algorithm’s optimization accuracy and convergence speed. However, when the algorithm deals with high-dimensional multi-modal problems, it is easy to fall into local optimum and barely obtain an ideal solution. In order to improve this situation, this paper designs a local optimal escape mechanism by combining chaos theory and opposition-based learning.

In recent years, many chaotic maps have been discovered and applied to various fields of human activities^26^. In intelligent algorithms, the chaotic map is widely used in population initialization, individual selection, mutation, and other processes^27,28^. Because the chaotic solution has the characteristics of ergodicity, randomness, and long-term unpredictability, it can achieve better results than random numbers and improve the algorithm’s performance.

In most existing literature, the application of chaotic maps commonly replaces Gaussian mutation, and the properties of chaotic solutions can be used to increase the diversity of the population. However, the unpredictable mutation of the chaotic step makes it difficult to adjust the step value according to the current population distribution. Thus, the algorithm’s further potential can not be exploited. Since the difference step has remarkable population adaptability, this paper creatively combines the difference step with the chaotic step and, at the same time, retains the advantages of the difference step and the chaotic step, i.e. it has the characteristics of timely adjusting the step with the distribution of the population and increasing the diversity of the population at the same time.

**Figure 2:**
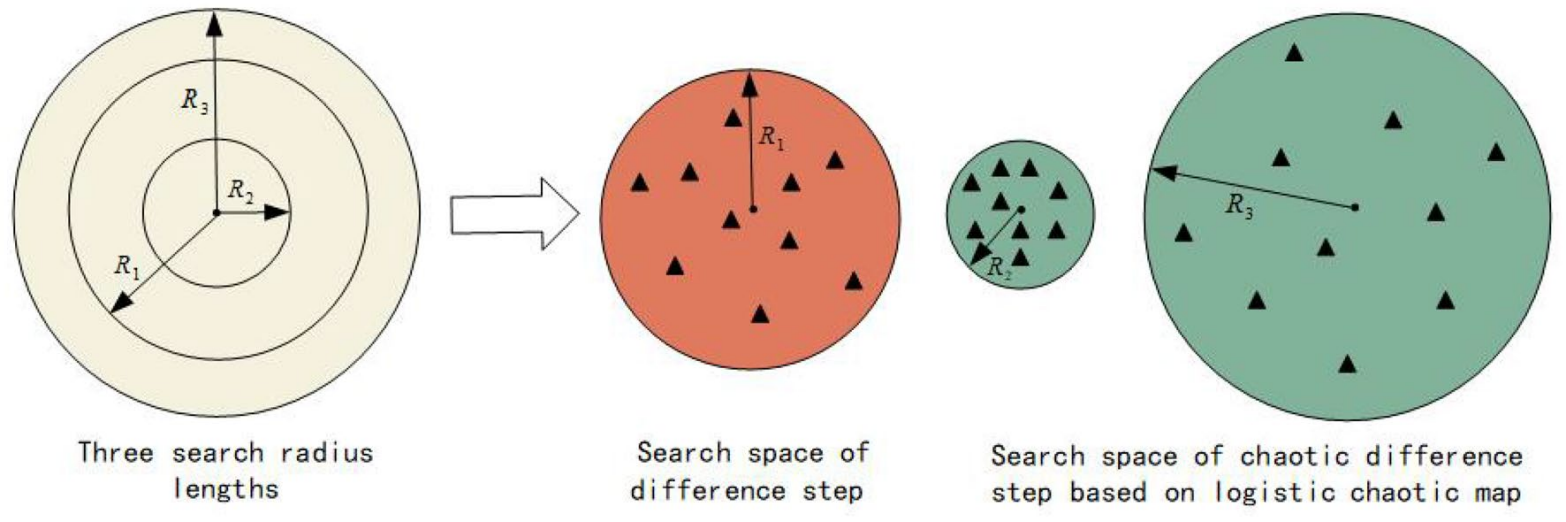
Schematic diagram of the chaotic difference step.

The chaotic difference step scales the difference step by introducing chaotic maps, which can expand the search space and reduce the time of falling into the local optimum. The schematic diagram of its function is shown in Fig. 2.

The chaotic difference step can be expressed as:

9 $$\begin{aligned} {X_m} = {X_s} + ({X_1} - {X_2}) \times rand() + ({X_1} - {X_2}) \times (y(t) - 0.5), \end{aligned}$$

where *y*(*t*) represents a chaotic map. The chaotic difference step formula and the schematic diagram are combined, which can reflect the superiority of the chaotic difference step. In Fig. 2, $R_1$ represents the average radius of the search space formed by the traditional difference step, $R_2$ and $R_3$ represent the average radius of the search space formed by the difference step after the perturbation of the logistic chaotic map. Two kinds of radii are formed because the logistic chaotic map has the characteristic that most of the solutions are distributed near 0 and 1. Since there is $(y(t) - 0.5)$ part in the formula, there will be two directions of radius reduction and increase to generate two search spaces. Take the population size of 10 as an example. It can be seen from Fig. 2 that the reduction of the search space makes the population more likely to find better solutions near the local optimal point in the range. At the same time, expanding the search space gives the population better diversity, which can make the positions of new individuals far away from the local optimum, increasing the possibility of jumping out of the local optimum.

Additionally, the solutions of different chaotic maps have different distribution characteristics. To fully expand the search space, various chaotic maps with different distributions and complementary chaotic maps are selected to form chaotic difference step with different intervals. Then, compare the fitness of the individuals after each chaotic difference mutation and select the best to retain and update the population so that the algorithm can jump out of the local optimum. The four chaotic maps selected in this paper are shown in the following Table 1:

**Table 1:** Definition of chaotic maps.

Chaotic map	Definition
Cubic	$y(t + 1) = \rho y(t)(1 - {y^2}(t))$
Sine	$y(t + 1) = \mu \sin (\pi y(t))$
Logistic	$y(t + 1) = \mu y(t)(1 - y(t))$
Circle	$y(t + 1) = \bmod (y(t) + 0.2 - (\frac{{0.5}}{{2\pi }})\sin (2\pi y(t)),1)$

**Figure 3:**
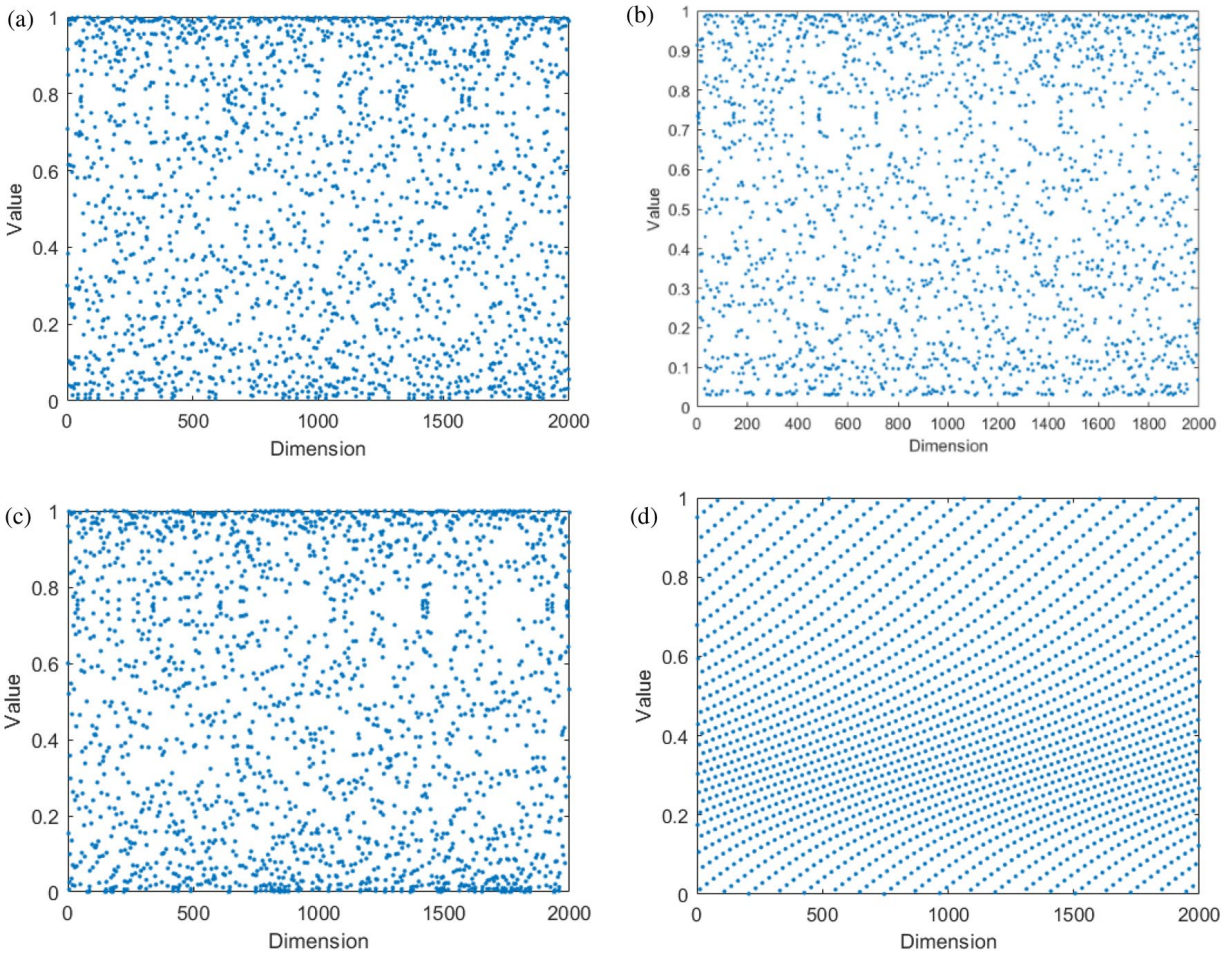
The distribution of the four chaotic maps.

We can achieve this better by illustrating the distinctions among each chaotic map through the visualization of chaotic maps^29^. Visualizations of four chaotic maps are presented in Fig. 3. Figure 3 illustrates the distribution of the chaotic maps. The four selected chaotic maps are distinguishable. The distribution complements each other, covering entirely between 0 and 1. The cubic map is evenly distributed and performs a thorough search across the entire interval from 0 to 1. The distribution of the sine map is similar to that of the Cubic map, but its properties lead to not being able to be distributed near the endpoint 0, thus extending the search space even more. The logistic map covers the full range from 0 to 1 but is more centered on the verge. Furthermore, it can cause a significant transformation of the step. The circle map shows a more even distribution within the range of 0.2 to 0.5, as evidenced by the distinct distribution displayed in the figure from the other three maps. Different distribution promotes the chaotic difference step adjustment and increases the algorithm’s ability to escape the local optimum.

There are various chaotic maps available in chaos theory, yet this work selects these four maps for two reasons. Firstly, more chaotic maps increase time complexity and hinder the algorithm’s overall performance. On the other hand, the four selected chaotic maps in this work are representative. While such map as the iterative map present a similar distribution to the logistic map, its introduction to the algorithm may fail to enhance its performance significantly. Therefore, these four chaotic maps are selected in this paper.

The chaotic difference step strategy can expand the search space of the population. Increasing search density improves the probability of jumping out of the local optimum if the search space is unchanged. Scientifically, opposition-based learning theory increases search density. Tizhoosh first proposed the idea of opposition-based learning and applied it to intelligence^30^. Then, numerous scholars applied this idea to the intelligent algorithms to improve the algorithms’ performance^31,32^. In these amounts of simulation experiments of the literature, generating opposition-based populations through opposition-based solutions can improve the performance of the algorithms. Compared to the existing literature on brain storm optimization algorithms, opposition-based learning is first introduced into the algorithm. Therefore, to further improve the ability of the algorithm to jump out of the local optimum, this paper breaks through and introduces the opposition-based learning thought into the brain storm optimization algorithm.

According to probability theory, a solution and its opposition-based solution have a half probability of achieving better results. Therefore, an opposition-based solution can be generated after the individual achieves mutation, and excellent individuals are retained to improve the probability of jumping out of the local optimum. The expression for the opposition-based solution is as follows:

10 $$\begin{aligned} X_o^d = Max{P^d} + Min{P^d} - X_m^d, \end{aligned}$$

where $X_o$ is the opposition-based solution, $X_o^d$ is the d-dimensional component of $X_o$, $Max{P^d}$ and $Min{P^d}$ represent the maximum and minimum values of the d-dimensional components of all individuals in the current population, $X_m^d$ is the d-dimension component of the outstanding individual retained by difference mutation and chaotic difference step.

**Figure 4:**
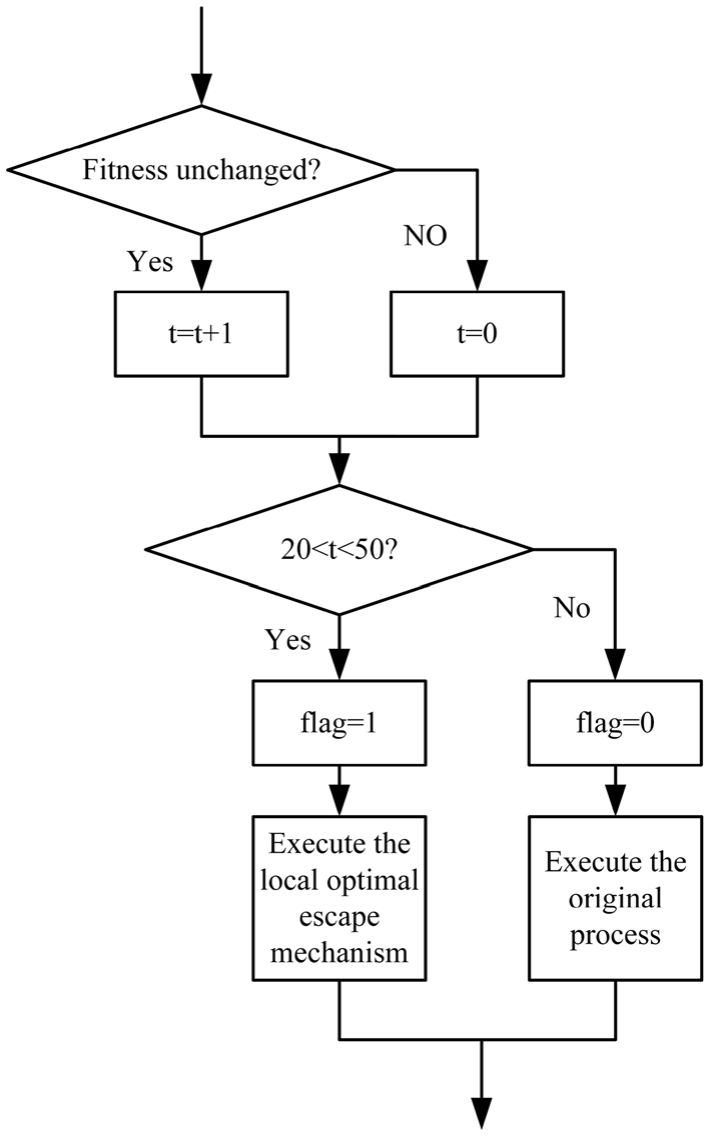
The trigger condition and end condition.

The chaotic difference step and the opposition-based population strategy do not conflict and jointly assist the algorithm in jumping out of the local optimum. However, it will significantly increase the time and space complexity of the algorithm. Therefore, a trigger condition and an end condition are required, and the schematic diagram is shown in Fig. 4.

Where *t* is the count variable, *flag* is the trigger variable. When *flag* is equal to 1, two strategies are executed, and when it is equal to 0, the original process remains unchanged. This design can improve the problem of increasing the algorithm’s time complexity caused by introducing a chaotic difference step and opposition-based population strategy in the COGBSO algorithm. Since the chaotic difference step and opposition-based population strategy in this paper are designed to assist the algorithm in jumping out of the local optimum, it is necessary to define when the algorithm is in the local optimum. After combining many experimental tests, this paper briefly defines the local optimum. If the algorithm falls into a local optimum, which means that when the optimal fitness value remains unchanged for more than 20 iterations, the search space is expanded through the chaotic difference step, and the opposition-based population is generated to help the algorithm escape the local optimum. The algorithm’s time complexity increases considerably at this point, so it is necessary to set an end condition to enable the algorithm to stop using both strategies in time. The end condition can be divided into two cases. On the one hand, if the algorithm’s fitness can quickly develop in a better direction after introducing the two strategies, it is considered that the strategies have played a role in assisting the algorithm to jump out of the local optimum. Then, the two strategies can be deactivated. On the other hand, if the algorithm’s fitness does not change within a long iteration period after the introduction of the two strategies, the new strategy is considered to have lost its effect, or the algorithm’s arithmetic power reaches its limit, which makes it impossible to improve its performance. Then, the algorithm can stop using the two strategies promptly, thus reducing the algorithm’s time complexity. The end conditions are also clearly defined in Fig. 4. If the fitness value of the algorithm changes or the fitness value does not change in 50 iterations, it will jump back to the original mutation process to reduce the algorithm’s time complexity. The algorithm pseudo code is shown in the following Algorithm 2.

**Algorithm 2:**
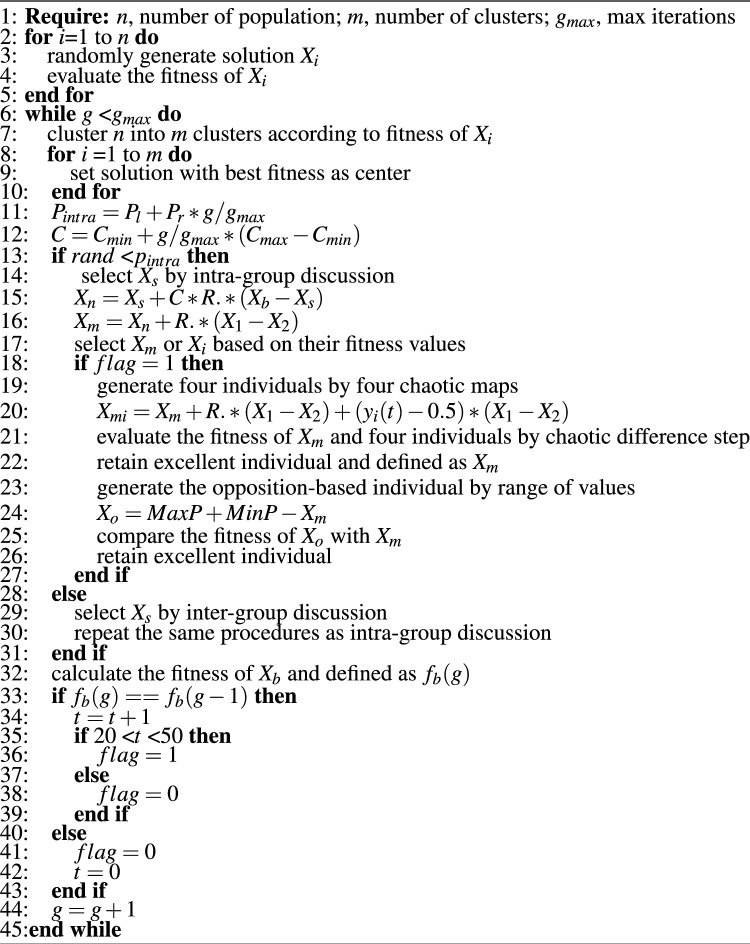
The COGBSO algorithm.

## Experimental results

According to the CEC2013 benchmark test suit, 15 benchmark functions, including uni-modal and multi-modal, are selected and defined in Table 2. Experiments are performed on 10, 20, and 30-dimensional conditions, respectively. Simulation comparison experiments are carried out for eight algorithms: BSO^5^, GBSO^15^, GDBSO^16^, ADMBSO^20^, SSA^33^, MSCA^34^, MSWOA^35^, and COGBSO. In addition, to more fully validate the performance of the algorithms, this paper also includes a variety of competitive intelligent algorithms tested using the CEC2018 benchmark test suit for comparison, with the test conditions set to 30-dimensional^9,36^. Then, analyze the performance of each algorithm, and each group of simulations is run 30 times independently. The simulation platform is Matlab 2018a.

**Table 2:** Benchmark functions.

Function	Definition	Range
F1	${F1}\mathrm{{ = }}\sum \limits _{i = 1}^D {x_i^2}$	[− 100, 100]
F2	${F2}\mathrm{{ = }}\sum \limits _{i = 1}^D {{{({{10}^6})}^{\frac{{i - 1}}{{D - 1}}}}} x_i^2$	[− 100, 100]
F3	$F3 = x_1^2 + {10^6}\sum \limits _{i = 2}^D {x_i^2}$	[− 100, 100]
F4	$F4 = {10^6}x_1^2 + \sum \limits _{i = 2}^D {x_i^2}$	[− 100, 100]
F5	$F5 = \sqrt{\sum \limits _{i = 1}^D {{|{{x_i}} |}^{2 + 4\frac{{i - 1}}{{D - 1}}}} }$	[− 100, 100]
F6	$F6 = \sum \limits _{i = 1}^{D - 1} {\left( {100{{\left( {x_i^2 - {x_{i + 1}}} \right) }^2} + {{\left( {{x_i} - 1} \right) }^2}} \right) }$	[− 2.048, 2.048]
F7	$F7 = 20 + e - 20\exp \left( { - 0.2\sqrt{\frac{1}{D}\sum \limits _{i = 1}^D {x_i^2} } } \right) + \exp \left( {\frac{1}{D}\sum \limits _{i = 1}^D {\cos \left( {2\pi {x_i}} \right) } } \right)$	[− 32, 32]
F8	$F8 = \sum \limits _{i = 1}^D {|{{x_i}\sin \left( {{x_i}} \right) + 0.1{x_i}} |}$	[− 10, 10]
F9	$F9 = \sum \limits _{i = 1}^D {\frac{{x_i^2}}{{4000}}} - \prod \limits _{i = 1}^D {\cos \left( {\frac{{{x_i}}}{{\sqrt{i} }}} \right) } + 1$	[− 600, 600]
F10	$F10 = \sum \limits _{i = 1}^D {\left( {x_i^2 - 10\cos \left( {2\pi {x_i}} \right) + 10} \right) }$	[− 5.12, 5.12]
F11	$F11 = \sum \limits _{i = 1}^D {|{{x_i}} |} + \prod \limits _{i = 1}^D {|{{x_i}} |}$	[− 100, 100]
F12	$F12 = \max |{{x_i}} |,(1< i < D)$	[− 100, 100]
F13	$F13 = \sum \limits _{i = 1}^D {x_i^{10}}$	[− 10, 10]
F14	$F14 = \sum \limits _{i = 1}^D {|{{x_i}} |}$	[− 100, 100]
F15	$F15 = \sum \limits _{i = 2}^D {\left( {{{\left( {{x_i} - 1} \right) }^2} + {{\left( {{x_1} - x_i^2} \right) }^2}} \right) }$	[0, 10]

### Parameters settings

Some basic parameters of the BSO algorithm and its improved algorithm compared in this paper are referenced in^5^. The parameter settings are as follows: the population size $n = 100$, the number of clusters $m = 5$, the adjustment factor $k = 20$, the number of evaluations ${F_{max}} = D*{10^4}$, the probability parameter ${P_{replace}} = 0.1$, ${P_{one}} = 0.5$, ${P_{one\_center}} = 0.3$ and ${P_{two\_center}} = 0.2$. The parameters (El-Abd, 2017) related to the global-best strategy are set as: ${C_{min}} = 0.2$ and ${C_{max}} = 0.8$. The discussion mechanism probability parameter (Yang et al.^20^) is set to ${P_l} = 0.2$ and ${P_r} = 0.7$. Meanwhile, the algorithm in this paper also compares five other types of swarm intelligence algorithms with parameter settings^9,33–36^.

### Simulation results and analysis

The optimization situation of each algorithm under the condition that the dimension *D* is equal to 10, 20, and 30 is shown in Tables 3, 4, 5, 6, 7, and 8. Each benchmark function is run 30 times, and four data types are obtained through statistical analysis: mean value, standard deviation, best value, and worst value. The comparison of the algorithms based on the complete CEC2018 benchmark test suit is shown in Tables 9 and 10. The best mean value of each benchmark function is marked in bold.

**Table 3:** Comparison between BSO variants on 10-D problems.

BFs		BSO	GBSO	ADMBSO	GDBSO	COGBSO
F1	Mean	2.17E-22	1.69E−22	7.27E−38	3.13E−39	**1.57E−67**
	Std	8.50E−23	7.54E−23	1.45E−37	5.81E−39	2.37E−67
	Min	5.16E−23	5.57E−23	8.41E−40	5.56E−41	3.34E−69
	Max	3.76E−22	3.76E−22	5.55E−37	2.06E−38	8.80E−67
F2	Mean	1.84E+04	2.49E−18	5.00E−33	5.06E−31	**3.86E−55**
	Std	2.24E+04	1.30E−18	1.19E−32	1.22E−30	1.18E−54
	Min	8.99E+01	1.16E−18	2.14E−35	4.83E−35	6.71E−58
	Max	8.62E+04	5.12E−18	4.67E−32	4.48E−30	4.64E−54
F3	Mean	2.35E+02	1.31E−16	2.10E−27	7.15E−24	**2.30E−42**
	Std	2.38E+02	4.35E−17	3.81E−27	2.63E−23	8.70E−42
	Min	1.38E−01	5.39E−17	2.55E−29	5.90E−28	3.16E−47
	Max	7.00E+02	2.14E−16	1.36E−26	1.02E−22	3.38E−41
F4	Mean	1.13E+03	3.18E−20	1.71E−36	5.34E−38	**3.37E−66**
	Std	8.45E+02	2.13E−20	1.93E−36	5.12E−38	6.91E−66
	Min	2.30E+02	1.05E−20	1.38E−38	3.71E−39	9.80E−69
	Max	3.42E+03	8.76E−20	7.38E−36	1.73E−37	2.69E−65
F5	Mean	8.65E−05	7.01E−14	5.55E−26	3.49E−25	**4.99E−38**
	Std	6.80E−05	3.97E−14	9.69E−26	7.51E−25	7.29E−38
	Min	1.03E−06	2.14E−14	1.28E−29	1.89E−28	9.06E−40
	Max	2.27E−04	1.52E−13	3.53E−25	2.68E−24	2.22E−37
F6	Mean	6.19E+00	5.38E+00	4.69E−08	5.57E−09	**6.17E−10**
	Std	1.07E+00	1.95E+00	5.34E−08	6.41E−09	4.60E−10
	Min	3.91E+00	2.00E−09	1.16E−10	6.36E−10	1.50E−10
	Max	8.22E+00	7.39E+00	1.43E−07	1.96E−08	1.08E−09
F7	Mean	1.88E−11	1.55E−11	2.93E−15	2.93E−15	**1.87E−15**
	Std	3.56E−12	3.20E−12	6.88E−16	6.88E−16	1.38E−15
	Min	1.36E−11	7.65E−12	4.44E−16	4.44E−16	4.44E−16
	Max	2.50E−11	1.92E−11	3.11E−15	3.11E−15	3.11E−15
F8	Mean	2.97E−03	3.71E−12	5.71E−09	1.68E−11	**6.05E−18**
	Std	2.39E−03	8.06E−13	2.11E−08	4.10E−11	1.61E−17
	Min	3.79E−12	2.50E−12	4.48E−15	2.91E−15	1.25E−33
	Max	7.31E−03	5.31E−12	8.18E−08	1.26E−10	5.15E−17
F9	Mean	4.30E+00	1.13E−01	1.38E−01	3.41E−01	**5.64E−02**
	Std	1.86E+00	7.84E−02	1.38E−01	1.27E−01	3.48E−02
	Min	2.57E+00	2.95E−02	1.48E−02	4.94E−02	9.86E−03
	Max	7.70E+00	2.61E−01	4.85E−01	5.26E−01	1.21E−01
F10	Mean	5.97E+00	4.78E+00	1.03E+01	2.14E+01	**4.31E+00**
	Std	1.88E+00	1.20E+00	6.45E+00	6.93E+00	2.15E+00
	Min	2.98E+00	2.98E+00	1.99E+00	4.98E+00	9.95E−01
	Max	8.95E+00	7.96E+00	2.39E+01	3.04E+01	6.96E+00
F11	Mean	3.57E+01	3.30E−11	6.36E−12	5.37E−12	**3.37E−23**
	Std	6.13E+01	9.03E−12	1.75E−11	1.99E−11	7.75E−23
	Min	3.65E−11	1.23E−11	1.12E−16	2.51E−16	1.33E−33
	Max	1.52E+02	4.60E−11	6.42E−11	7.72E−11	2.97E−22
F12	Mean	9.14E−12	7.51E−12	8.95E−15	1.88E−15	**3.76E−23**
	Std	1.72E−12	1.09E−12	5.57E−15	1.67E−15	3.76E−23
	Min	6.09E−12	5.45E−12	1.42E−15	2.60E−16	4.06E−24
	Max	1.18E−11	9.12E−12	2.03E−14	6.39E−15	1.46E−22
F13	Mean	1.45E−110	4.33E−111	3.71E−157	4.73E−168	**1.48E−267**
	Std	1.75E−110	6.41E−111	0.00E+00	0.00E+00	0.00E+00
	Min	1.12E−111	1.18E−113	3.64E−169	1.21E−178	2.43E−283
	Max	6.72E−110	2.43E−110	3.23E−156	6.47E−167	1.11E−266
F14	Mean	4.06E−11	3.12E−11	1.32E−17	3.74E−18	**3.71E−25**
	Std	5.91E−12	5.16E−12	1.06E−17	3.92E−18	4.45E−25
	Min	3.17E−11	2.53E−11	1.27E−18	4.24E−19	1.89E−26
	Max	5.05E−11	4.45E−11	4.10E−17	1.47E−17	1.67E−24
F15	Mean	1.17E−11	9.62E−22	**0.00E+00**	**0.00E+00**	**0.00E+00**
	Std	4.55E−11	2.18E−22	0.00E+00	0.00E+00	0.00E+00
	Min	1.01E−21	5.95E−22	0.00E+00	0.00E+00	0.00E+00
	Max	1.76E−10	1.36E−21	0.00E+00	0.00E+00	0.00E+00

Optimal mean values are in bold.

**Table 4:** Comparison between BSO variants on 20-D problems.

BFs		BSO	GBSO	ADMBSO	GDBSO	COGBSO
F1	Mean	3.31E−43	1.56E−43	2.89E−50	9.73E−51	**8.07E−66**
	Std	9.04E−44	3.34E−44	3.69E−50	3.49E−50	1.87E−65
	Min	1.95E−43	1.09E−43	2.73E−52	1.36E−54	8.53E−69
	Max	5.15E−43	2.20E−43	1.30E−49	1.36E−49	7.12E−65
F2	Mean	9.36E+04	2.07E−39	7.98E−45	2.59E−45	**1.10E−64**
	Std	4.97E+04	1.06E−39	1.27E−44	2.82E−45	1.88E−64
	Min	2.53E+04	8.94E−40	2.47E−46	8.16E−48	1.61E−67
	Max	2.14E+05	4.80E−39	3.99E−44	8.36E−45	6.33E−64
F3	Mean	4.08E+02	6.64E−34	5.39E−34	1.91E−32	**3.11E−55**
	Std	5.66E+02	2.08E−33	1.53E−33	5.19E−32	7.43E−55
	Min	3.76E+00	1.74E−37	6.39E−36	2.56E−37	4.17E−59
	Max	2.05E+03	8.09E−33	6.02E−33	1.98E−31	2.19E−54
F4	Mean	5.28E+02	1.44E−41	2.11E−49	3.71E−51	**3.48E−66**
	Std	3.13E+02	1.14E−41	3.16E−49	5.93E−51	8.20E−66
	Min	3.20E+01	1.58E−42	3.25E−52	9.64E−54	1.16E−69
	Max	1.03E+03	3.74E−41	1.22E−48	2.28E−50	3.23E−65
F5	Mean	1.41E−03	1.28E−24	5.37E−37	1.98E−38	**2.09E−49**
	Std	4.97E−04	5.74E−25	1.75E−36	4.26E−38	3.46E−49
	Min	6.07E−04	4.20E−25	9.23E−41	1.90E−43	2.18E−52
	Max	2.51E−03	2.34E−24	6.85E−36	1.43E−37	1.00E−48
F6	Mean	1.77E+01	1.67E+01	2.66E−01	2.41E−07	**5.43E−08**
	Std	9.01E−01	4.70E+00	1.03E+00	3.52E−07	2.15E−08
	Min	1.60E+01	1.31E−07	1.03E−07	4.22E−08	2.09E−08
	Max	1.92E+01	1.93E+01	3.99E+00	1.41E−06	8.46E−08
F7	Mean	3.64E−15	**2.22E−15**	2.46E−15	**2.22E−15**	**2.22E−15**
	Std	1.80E−15	8.17E−31	9.17E−16	8.17E−31	8.17E−31
	Min	2.22E−15	2.22E−15	2.22E−15	2.22E−15	2.22E−15
	Max	5.77E−15	2.22E−15	5.77E−15	2.22E−15	2.22E−15
F8	Mean	6.81E−02	8.14E−17	5.71E−17	1.22E−16	**1.48E−17**
	Std	6.97E−02	2.15E−16	1.63E−16	2.53E−16	5.73E−17
	Min	1.42E−02	3.63E−23	1.30E−21	2.93E−21	1.02E−39
	Max	2.76E−01	6.11E−16	6.11E−16	6.11E−16	2.22E−16
F9	Mean	2.35E−02	8.21E−03	8.54E−03	7.88E−03	**1.31E−03**
	Std	2.27E−02	1.09E−02	6.37E−03	9.36E−03	5.09E−03
	Min	0.00E+00	0.00E+00	0.00E+00	0.00E+00	0.00E+00
	Max	7.14E−02	2.96E−02	1.97E−02	2.95E−02	1.97E−02
F10	Mean	1.92E+01	1.30E+01	3.10E+01	5.02E+01	**1.05E+01**
	Std	4.40E+00	5.33E+00	2.71E+01	3.82E+01	3.94E+00
	Min	1.29E+01	4.97E+00	6.96E+00	5.97E+00	2.89E+00
	Max	2.89E+01	2.39E+01	8.95E+01	1.05E+02	1.89E+01
F11	Mean	2.20E+02	2.36E−21	2.33E−17	1.28E−17	**3.70E−36**
	Std	5.76E+01	3.95E−22	4.40E−17	4.88E−17	7.08E−36
	Min	9.75E+01	1.82E−21	1.75E−19	4.93E−21	1.34E−46
	Max	3.17E+02	3.01E−21	1.52E−16	1.89E−16	2.15E−35
F12	Mean	1.20E−03	**2.05E−17**	6.05E−15	5.91E−16	2.72E−16
	Std	1.46E−03	2.30E−17	6.08E−15	4.95E−16	4.11E−16
	Min	6.47E−07	4.08E−19	1.00E−15	5.54E−17	2.28E−17
	Max	4.43E−03	7.74E−17	2.24E−14	1.71E−15	1.59E−15
F13	Mean	3.51E−45	**2.11E−212**	6.03E−180	4.58E−192	1.58E−211
	Std	1.36E−44	0.00E+00	0.00E+00	0.00E+00	0.00E+00
	Min	2.56E−216	7.00E−216	4.79E−198	1.05E−206	2.32E−223
	Max	5.26E−44	3.00E−211	9.04E−179	4.06E−191	2.01E−210
F14	Mean	2.69E−09	1.37E−21	7.89E−23	8.46E−24	**8.69E−34**
	Std	1.04E−08	1.54E−22	5.82E−23	1.02E−23	1.55E−33
	Min	1.67E−21	1.17E−21	1.08E−23	7.67E−25	5.97E−35
	Max	4.03E−08	1.73E−21	1.94E−22	2.99E−23	6.28E−33
F15	Mean	5.57E−03	**0.00E+00**	**0.00E+00**	**0.00E+00**	1.04E−30
	Std	4.88E−03	0.00E+00	0.00E+00	0.00E+00	1.12E−30
	Min	1.13E−03	0.00E+00	0.00E+00	0.00E+00	0.00E+00
	Max	1.87E−02	0.00E+00	0.00E+00	0.00E+00	4.19E−30

Optimal mean values are in bold.

**Table 5:** Comparison between BSO variants on 30-D problems.

BFs		BSO	GBSO	ADMBSO	GDBSO	COGBSO
F1	Mean	2.00E−64	7.02E−65	9.20E−63	3.02E−67	**5.40E−78**
	Std	4.19E−65	1.70E−65	2.00E−62	4.00E−67	1.06E−77
	Min	1.28E−64	5.12E−65	1.84E−65	7.58E−69	5.03E−80
	Max	2.53E−64	1.00E−64	6.38E−62	9.09E−67	3.45E−77
F2	Mean	2.19E+05	8.69E−58	1.04E−54	1.25E−57	**3.47E−79**
	Std	9.97E+04	1.86E−57	2.75E−54	2.30E−57	8.70E−79
	Min	9.60E+04	2.64E−60	2.20E−58	1.36E−60	7.15E−83
	Max	4.08E+05	7.13E−57	1.05E−53	8.74E−57	3.27E−78
F3	Mean	3.79E+02	1.15E−42	1.07E−41	4.17E−41	**2.25E−67**
	Std	6.13E+02	2.75E−42	1.89E−41	1.02E−40	6.44E−67
	Min	3.37E−02	2.88E−47	5.12E−44	3.98E−45	8.86E−72
	Max	2.31E+03	9.03E−42	5.98E−41	3.94E−40	2.41E−66
F4	Mean	2.85E+02	3.47E−63	8.08E−63	5.04E−66	**3.18E−77**
	Std	2.62E+02	2.84E−63	9.66E−63	5.82E−66	8.82E−77
	Min	3.86E+01	8.43E−64	1.72E−65	8.21E−68	8.31E−80
	Max	8.42E+02	1.13E−62	3.12E−62	1.85E−65	3.48E−76
F5	Mean	5.03E−03	1.58E−35	3.02E−46	2.41E−49	**2.01E−58**
	Std	1.44E−03	5.79E−36	3.20E−46	6.49E−49	5.05E−58
	Min	3.23E−03	7.12E−36	8.79E−49	4.14E−53	8.26E−62
	Max	8.10E−03	2.38E−35	9.03E−46	2.50E−48	1.99E−57
F6	Mean	2.84E+01	1.78E+01	5.32E−01	8.95E−06	**6.36E−06**
	Std	5.30E−01	1.75E+01	1.40E+00	1.20E−05	9.87E−06
	Min	2.77E+01	9.33E−07	5.49E−06	9.71E−07	5.41E−07
	Max	2.94E+01	3.92E+01	3.99E+00	4.52E−05	2.83E−05
F7	Mean	1.28E−14	3.82E−15	5.71E−15	5.48E−15	**3.35E−15**
	Std	3.41E−15	1.47E−15	1.63E−15	1.73E−15	9.17E−16
	Min	6.66E−15	3.11E−15	3.11E−15	3.11E−15	3.11E−15
	Max	2.09E−14	6.66E−15	6.66E−15	6.66E−15	6.66E−15
F8	Mean	3.23E−01	4.66E−16	4.48E−16	3.77E−16	**1.11E−16**
	Std	3.07E−01	6.81E−16	4.32E−16	6.07E−16	2.33E−16
	Min	3.45E−02	1.01E−33	1.74E−29	1.21E−25	4.77E−43
	Max	1.02E+00	2.04E−15	1.22E−15	2.11E−15	6.11E−16
F9	Mean	8.55E−03	6.18E−03	4.44E−03	4.60E−03	**8.21E−04**
	Std	9.15E−03	6.90E−03	4.49E−03	6.83E−03	3.18E−03
	Min	5.76E−07	0.00E+00	0.00E+00	0.00E+00	0.00E+00
	Max	3.20E−02	2.22E−02	1.23E−02	2.22E−02	1.23E−02
F10	Mean	3.67E+01	3.47E+01	3.04E+01	4.14E+01	**1.70E+01**
	Std	6.46E+00	8.90E+00	8.23E+00	4.22E+01	5.51E+00
	Min	2.79E+01	1.92E+01	1.79E+01	1.49E+01	9.95E+00
	Max	4.78E+01	4.95E+01	4.18E+01	1.45E+02	2.79E+01
F11	Mean	3.79E+02	6.11E−25	5.57E−23	1.40E−23	**7.95E−40**
	Std	9.30E+01	2.35E−24	1.92E−22	5.36E−23	3.08E−39
	Min	2.31E+02	3.04E−30	4.79E−26	5.69E−28	2.69E−47
	Max	5.90E+02	9.09E−24	7.48E−22	2.08E−22	1.19E−38
F12	Mean	4.67E−02	**2.13E−17**	1.05E−13	1.07E−16	4.67E−15
	Std	1.50E−02	2.91E−17	1.22E−13	1.49E−16	2.74E−15
	Min	2.15E−02	5.50E−18	8.10E−15	2.23E−17	7.76E−16
	Max	7.43E−02	1.09E−16	4.70E−13	5.21E−16	1.08E−14
F13	Mean	3.64E−20	**3.18E−245**	1.36E−216	1.94E−235	2.33E−203
	Std	8.13E−20	0.00E+00	0.00E+00	0.00E+00	0.00E+00
	Min	1.55E−26	3.50E−263	7.11E−225	2.25E−252	1.43E−218
	Max	2.83E−19	3.05E−244	1.11E−215	2.86E−234	2.66E−202
F14	Mean	2.83E−02	2.23E−31	4.05E−29	3.65E−30	**1.83E−42**
	Std	3.24E−02	2.16E−31	5.95E−29	5.15E−30	4.60E−42
	Min	2.38E−04	6.45E−32	1.34E−30	2.00E−31	8.36E−45
	Max	1.07E−01	6.74E−31	2.09E−28	1.85E−29	1.82E−41
F15	Mean	1.72E−01	**0.00E+00**	7.48E−32	**0.00E+00**	1.54E−29
	Std	7.65E−02	0.00E+00	1.62E−31	0.00E+00	1.76E−29
	Min	5.32E−02	0.00E+00	0.00E+00	0.00E+00	1.43E−30
	Max	2.88E−01	0.00E+00	4.93E−31	0.00E+00	6.54E−29

Optimal mean values are in bold.

**Table 6:** Comparison between different swarm intelligence algorithms on 10-D problems.

BFs		SSA	MSWOA	MSCA	COGBSO
F1	Mean	5.94E−10	2.39E−67	1.85E−51	**1.57E−67**
	Std	1.81E−10	1.44E−67	5.14E−51	2.37E−67
	Min	3.76E−10	1.18E−67	2.67E−70	3.34E−69
	Max	9.73E−10	4.54E−67	1.81E−50	8.80E−67
F2	Mean	9.59E+02	1.07E−54	1.81E−50	**3.86E−55**
	Std	6.80E+02	2.00E−54	6.94E−50	1.18E−54
	Min	1.44E+02	1.21E−55	7.93E−67	6.71E−58
	Max	2.37E+03	3.87E−54	2.69E−49	4.64E−54
F3	Mean	4.03E+00	6.59E−39	1.59E−40	**2.30E−42**
	Std	4.87E+00	1.02E−39	6.17E−40	8.70E−42
	Min	3.45E−02	9.88E−48	2.56E−47	3.16E−47
	Max	1.80E+01	3.48E−37	2.39E−39	3.38E−41
F4	Mean	1.89E+02	2.77E−62	1.04E−53	**3.37E−66**
	Std	2.30E+02	5.48E−62	2.43E−53	6.91E−66
	Min	9.14E+00	5.12E−68	9.56E−73	9.80E−69
	Max	6.87E+02	3.78E−61	9.12E−53	2.69E−65
F5	Mean	1.41E−04	**0.00E+00**	1.76E−28	4.99E−38
	Std	4.33E−05	0.00E+00	4.65E−28	7.29E−38
	Min	7.53E−05	0.00E+00	2.92E−30	9.06E−40
	Max	2.55E−04	0.00E+00	1.53E−27	2.22E−37
F6	Mean	4.58E−08	1.20E+00	6.47E+00	**6.17E−10**
	Std	1.04E−08	2.10E+00	3.89E−01	4.60E−10
	Min	4.20E−08	1.70E−07	6.15E+00	1.50E−10
	Max	4.60E−08	5.96E+00	7.22E+00	1.08E−09
F7	Mean	2.38E−01	**− 4.44E−16**	**− 4.44E−16**	1.87E−15
	Std	2.38E−01	− 4.44E−16	− 4.44E−16	1.38E−15
	Min	5.12E−06	− 4.44E−16	− 4.44E−16	4.44E−16
	Max	3.57E+00	− 4.44E−16	− 4.44E−16	3.11E−15
F8	Mean	2.20E−05	3.87E−05	**1.86E−28**	6.05E−18
	Std	6.21E−06	1.50E−04	7.20E−28	1.61E−17
	Min	1.55E−05	1.52E−17	2.91E−40	1.25E−33
	Max	4.22E−05	5.80E−04	2.79E−27	5.15E−17
F9	Mean	1.62E−02	**0.00E+00**	**0.00E+00**	5.64E−02
	Std	3.87E−02	0.00E+00	0.00E+00	3.48E−02
	Min	1.16E−09	0.00E+00	0.00E+00	9.86E−03
	Max	1.35E−01	0.00E+00	0.00E+00	1.21E−01
F10	Mean	9.95E+00	5.62E+00	9.95E+00	**4.31E+00**
	Std	8.50E−11	3.33E+00	9.95E+00	2.15E+00
	Min	9.95E+00	9.95E−01	9.95E+00	9.95E−01
	Max	9.95E+00	6.96E+00	9.95E+00	6.96E+00
F11	Mean	6.19E−05	6.41E−17	3.02E−11	**3.37E−23**
	Std	8.24E−06	8.85E−17	1.10E−10	7.75E−23
	Min	4.33E−05	6.86E−18	1.39E−19	1.33E−33
	Max	7.46E−05	9.02E−17	4.27E−10	2.97E−22
F12	Mean	1.31E−05	7.20E−19	4.55E−18	**3.76E−23**
	Std	1.67E−06	6.51E−19	1.76E−17	3.76E−23
	Min	9.40E−06	6.26E−20	5.56E−28	4.06E−24
	Max	1.48E−05	2.10E−13	6.83E−17	1.46E−22
F13	Mean	1.40E−58	**0.00E+00**	3.25E−229	1.48E−267
	Std	2.25E−58	0.00E+00	0.00E+00	0.00E+00
	Min	8.32E−61	0.00E+00	1.36E−317	2.43E−283
	Max	7.85E−58	0.00E+00	4.76E−228	1.11E−266
F14	Mean	5.94E−05	**2.94E−179**	9.70E−28	3.71E−25
	Std	1.13E−05	0.00E+00	3.73E−27	4.45E−25
	Min	3.89E−05	3.59E−182	7.51E−41	1.89E−26
	Max	7.54E−05	2.50E−178	1.45E−26	1.67E−24
F15	Mean	9.12E−13	1.10E−01	4.38E+00	**0.00E+00**
	Std	6.16E−19	4.20E−01	1.67E+00	0.00E+00
	Min	9.12E−13	5.27E−04	4.72E−01	0.00E+00
	Max	9.12E−13	1.63E+00	6.16E+00	0.00E+00

Optimal mean values are in bold.

**Table 7:** Comparison between different swarm intelligence algorithms on 20-D problems.

BFs		SSA	MSWOA	MSCA	COGBSO
F1	Mean	2.43E−09	2.55E−65	1.73E−64	**8.07E−66**
	Std	4.40E−10	6.17E−66	6.69E−64	1.87E−65
	Min	1.84E−09	1.05E−66	2.46E−65	8.53E−69
	Max	3.35E−09	1.59E−65	2.59E−63	7.12E−65
F2	Mean	3.58E+03	2.70E−63	8.64E−63	**1.10E−64**
	Std	2.62E+03	4.91E−64	3.35E−62	1.88E−64
	Min	4.60E+02	1.03E−64	2.67E−79	1.61E−67
	Max	1.06E+04	1.33E−63	1.30E−61	6.33E−64
F3	Mean	6.67E+00	4.60E−54	5.25E−49	**3.11E−55**
	Std	6.62E+00	1.04E−54	2.03E−48	7.43E−55
	Min	1.44E−01	3.38E−55	2.09E−57	4.17E−59
	Max	1.92E+01	2.19E−54	7.88E−48	2.19E−54
F4	Mean	3.81E+01	9.85E−66	6.44E−64	**3.48E−66**
	Std	2.45E+01	1.26E−65	8.33E−64	8.20E−66
	Min	1.02E+01	2.64E−66	4.32E−65	1.16E−69
	Max	1.12E+02	3.23E−65	5.16E−63	3.23E−65
F5	Mean	3.83E−04	**0.00E+00**	4.88E−48	2.09E−49
	Std	8.33E−05	0.00E+00	1.39E−47	3.46E−49
	Min	2.02E−04	0.00E+00	1.06E−51	2.18E−52
	Max	4.84E−04	0.00E+00	5.07E−47	1.00E−48
F6	Mean	9.72E−11	2.93E−01	1.69E+01	**5.43E−08**
	Std	3.16E−18	1.07E+00	6.05E−01	2.15E−08
	Min	9.72E−11	1.33E−09	1.61E+01	2.09E−08
	Max	9.72E−11	4.14E+00	1.80E+01	8.46E−08
F7	Mean	3.57E+00	**−1.33E−15**	**−1.33E−15**	2.22E−15
	Std	0.00E+00	0.00E+00	0.00E+00	8.17E−31
	Min	3.57E+00	−1.33E−15	−1.33E−15	2.22E−15
	Max	3.57E+00	−1.33E−15	−1.33E−15	2.22E−15
F8	Mean	6.28E−05	3.88E−05	**1.33E−41**	1.48E−17
	Std	9.86E−06	1.50E−04	4.75E−41	5.73E−17
	Min	5.10E−05	0.00E+00	8.60E−67	1.02E−39
	Max	8.19E−05	5.83E−04	1.84E−40	2.22E−16
F9	Mean	6.86E−09	**0.00E+00**	**0.00E+00**	1.31E−03
	Std	1.93E−09	0.00E+00	0.00E+00	5.09E−03
	Min	3.43E−09	0.00E+00	0.00E+00	0.00E+00
	Max	1.02E−08	0.00E+00	0.00E+00	1.97E−02
F10	Mean	1.99E+01	1.27E+01	1.56E+01	**1.05E+01**
	Std	2.31E−10	3.61E+00	4.23E+00	3.94E+00
	Min	1.99E+01	1.89E+01	5.64E+00	2.89E+00
	Max	1.99E+01	9.95E+00	2.39E+01	1.89E+01
F11	Mean	1.92E−04	7.90E−32	5.63E−31	**3.70E−36**
	Std	2.04E−05	8.88E−32	2.13E−30	7.08E−36
	Min	1.68E−04	6.40E−32	2.25E−37	1.34E−46
	Max	2.32E−04	9.40E−32	8.26E−30	2.15E−35
F12	Mean	1.00E+00	7.10E−15	**3.75E−22**	2.72E−16
	Std	4.60E−16	1.02E−16	1.05E−21	4.11E−16
	Min	1.00E+00	4.77E−17	1.80E−38	2.28E−17
	Max	1.00E+00	1.98E−14	3.75E−21	1.59E−15
F13	Mean	3.05E−56	**0.00E+00**	1.35E−182	1.58E−211
	Std	4.14E−56	0.00E+00	0.00E+00	0.00E+00
	Min	3.90E−58	0.00E+00	3.36E−193	2.32E−223
	Max	1.55E−55	0.00E+00	2.03E−181	2.01E−210
F14	Mean	1.97E−04	**7.90E−223**	7.98E−40	8.69E−34
	Std	1.90E−05	0.00E+00	2.99E−39	1.55E−33
	Min	1.66E−04	4.40E−223	4.33E−55	5.97E−35
	Max	2.25E−04	8.90E−223	1.16E−38	6.28E−33
F15	Mean	1.92E−12	5.11E−01	1.37E+01	**1.04E−30**
	Std	6.77E−19	7.96E−01	2.75E+00	1.12E−30
	Min	1.92E−12	6.11E−03	4.15E+00	0.00E+00
	Max	1.92E−12	1.81E+00	1.60E+01	4.19E−30

Optimal mean values are in bold.

**Table 8:** Comparison between different swarm intelligence algorithms on 30-D problems.

BFs		SSA	MSWOA	MSCA	COGBSO
F1	Mean	5.84E−09	1.13E−77	5.16E−74	**5.40E−78**
	Std	1.03E−09	1.35E−77	2.00E−73	1.06E−77
	Min	4.31E−09	1.22E−78	7.04E−75	5.03E−80
	Max	7.57E−09	3.45E−77	7.74E−73	3.45E−77
F2	Mean	5.59E+03	1.02E−78	6.09E−76	**3.47E−79**
	Std	3.07E+03	1.34E−78	2.36E−75	8.70E−79
	Min	1.62E+03	1.55E−79	2.03E−84	7.15E−83
	Max	1.07E+04	5.36E−78	9.13E−75	3.27E−78
F3	Mean	1.26E+01	5.61E−67	1.35E−58	**2.25E−67**
	Std	1.78E+01	9.67E−67	5.24E−58	6.44E−67
	Min	6.52E−03	3.40E−69	4.27E−67	8.86E−72
	Max	6.16E+01	3.41E−66	2.03E−57	2.41E−66
F4	Mean	2.66E+01	3.73E−77	3.53E−75	**3.18E−77**
	Std	2.03E+01	1.33E−77	1.36E−74	8.82E−77
	Min	9.68E+00	1.64E−78	8.74E−82	8.31E−80
	Max	9.09E+01	1.02E−76	5.27E−74	3.48E−76
F5	Mean	5.62E−04	**0.00E+00**	2.33E−54	2.01E−58
	Std	6.51E−05	0.00E+00	9.03E−54	5.05E−58
	Min	4.58E−04	0.00E+00	2.39E−56	8.26E−62
	Max	6.74E−04	0.00E+00	3.50E−53	1.99E−57
F6	Mean	1.48E−10	1.07E+00	2.68E+01	**6.36E−06**
	Std	4.13E−18	2.32E+00	7.93E−01	9.87E−06
	Min	1.48E−10	3.05E−09	2.58E+01	5.41E−07
	Max	1.48E−10	7.76E+00	2.80E+01	2.83E−05
F7	Mean	3.57E+00	**− 4.44E−16**	**− 4.44E−16**	3.35E−15
	Std	1.23E−05	0.00E+00	0.00E+00	9.17E−16
	Min	3.57E+00	−4.44E−16	−4.44E−16	3.11E−15
	Max	3.57E+00	−4.44E−16	−4.44E−16	6.66E−15
F8	Mean	1.18E−04	9.10E−05	**2.52E−56**	1.11E−16
	Std	1.23E−05	2.37E−04	9.69E−56	2.33E−16
	Min	1.00E−04	0.00E+00	3.43E−70	4.77E−43
	Max	1.47E−04	8.91E−04	3.75E−55	6.11E−16
F9	Mean	1.31E−08	**0.00E+00**	**0.00E+00**	8.21E−04
	Std	3.30E−09	0.00E+00	0.00E+00	3.18E−03
	Min	9.17E−09	0.00E+00	0.00E+00	0.00E+00
	Max	2.06E−08	0.00E+00	0.00E+00	1.23E−02
F10	Mean	2.98E+01	1.83E+01	1.93E+01	**1.70E+01**
	Std	4.52E−10	6.10E+00	6.84E+00	5.51E+00
	Min	2.98E+01	9.95E+00	1.09E+01	9.95E+00
	Max	2.98E+01	2.29E+01	2.79E+01	2.79E+01
F11	Mean	3.51E−04	1.14E−34	1.12E−39	**7.95E−40**
	Std	3.69E−05	3.73E−34	4.31E−39	3.08E−39
	Min	2.88E−04	9.40E−35	7.64E−48	2.69E−47
	Max	4.34E−04	1.40E−33	1.67E−38	1.19E−38
F12	Mean	1.00E+00	4.90E−15	**2.46E−24**	4.67E−15
	Std	2.30E−16	0.00E+00	9.52E−24	2.74E−15
	Min	1.00E+00	4.90E−15	4.72E−34	7.76E−16
	Max	1.00E+00	4.90E−15	3.69E−23	1.08E−14
F13	Mean	6.25E−55	**0.00E+00**	**0.00E+00**	2.33E−203
	Std	7.53E−55	0.00E+00	0.00E+00	0.00E+00
	Min	7.62E−56	0.00E+00	0.00E+00	1.43E−218
	Max	2.86E−54	0.00E+00	0.00E+00	2.66E−202
F14	Mean	3.77E−04	**1.20E−272**	2.31E−49	1.83E−42
	Std	4.96E−05	0.00E+00	8.95E−49	4.60E−42
	Min	3.12E−04	9.40E−273	7.87E−58	8.36E−45
	Max	4.60E−04	1.40E−272	3.47E−48	1.82E−41
F15	Mean	2.94E−12	1.39E+00	2.38E+01	**1.54E−29**
	Std	8.86E−19	1.27E+00	1.12E+00	1.76E−29
	Min	2.94E−12	1.44E−02	2.20E+01	1.43E−30
	Max	2.94E−12	3.42E+00	2.62E+01	6.54E−29

Optimal mean values are in bold.

**Table 9:** Comparison of COGBSO with BSO variants for CEC 2018 test functions with D = 30.

BFs		BSO	GBSO	ADMBSO	GDBSO	COGBSO
F1	Mean	1.18E+08	3.10E+03	3.10E+03	**1.41E+03**	3.63E+03
	Std	1.97E+08	2.52E+03	2.52E+03	1.76E+03	4.25E+03
F3	Mean	8.92E+04	7.28E+04	3.94E+04	7.98E+04	**3.32E+02**
	Std	2.41E+04	1.88E+04	8.52E+03	1.25E+04	6.19E+01
F4	Mean	5.88E+02	4.98E+02	4.98E+02	**4.90E+02**	5.06E+02
	Std	5.67E+01	1.37E+01	1.32E+01	1.79E+01	1.17E+01
F5	Mean	6.82E+02	7.05E+02	5.60E+02	7.03E+02	**5.48E+02**
	Std	3.06E+01	1.62E+01	2.43E+01	1.71E+01	1.72E+01
F6	Mean	6.53E+02	6.00E+02	6.00E+02	6.01E+02	**6.00E+02**
	Std	7.45E+00	1.47E−01	4.37E−02	3.04E−01	5.47E−02
F7	Mean	1.12E+03	9.42E+02	**7.95E+02**	9.45E+02	8.13E+02
	Std	5.79E+01	1.18E+01	1.70E+01	1.38E+01	6.60E+01
F8	Mean	9.34E+02	1.00E+03	8.62E+02	1.01E+03	**8.39E+02**
	Std	2.53E+01	1.72E+01	1.82E+01	1.14E+01	9.46E+00
F9	Mean	3.73E+03	9.02E+02	**9.00E+02**	9.29E+02	9.04E+02
	Std	5.33E+02	2.10E+00	7.51E−01	7.64E+01	5.83E+00
F10	Mean	**4.91E+03**	8.55E+03	5.09E+03	8.59E+03	8.07E+03
	Std	5.22E+02	4.17E+02	4.20E+02	2.52E+02	2.93E+02
F11	Mean	1.38E+03	1.23E+03	1.17E+03	1.24E+03	**1.17E+03**
	Std	1.61E+02	3.39E+01	3.48E+01	2.68E+01	2.53E+01
F12	Mean	1.92E+07	1.08E+05	9.14E+04	8.91E+04	**3.87E+04**
	Std	1.89E+07	8.19E+04	7.16E+04	4.40E+04	1.84E+04
F13	Mean	3.79E+04	1.65E+04	1.53E+04	1.17E+04	**1.04E+04**
	Std	9.42E+03	1.33E+04	1.19E+04	1.16E+04	1.65E+04
F14	Mean	1.81E+05	4.92E+04	1.34E+04	1.41E+04	**2.41E+03**
	Std	2.16E+05	4.79E+04	2.17E+04	9.31E+03	9.15E+02
F15	Mean	3.17E+04	8.32E+03	5.10E+03	5.84E+03	**4.92E+03**
	Std	1.83E+04	7.09E+03	2.48E+03	3.72E+03	3.25E+03
F16	Mean	3.18E+03	3.25E+03	2.76E+03	3.25E+03	**2.14E+03**
	Std	2.55E+02	3.18E+02	3.02E+02	1.99E+02	1.63E+02
F17	Mean	2.36E+03	2.38E+03	2.05E+03	2.26E+03	**1.85E+03**
	Std	3.51E+02	1.23E+02	1.60E+02	2.18E+02	7.56E+01
F18	Mean	4.01E+05	2.10E+06	2.10E+05	1.33E+06	**7.93E+04**
	Std	3.11E+05	9.49E+05	1.34E+05	8.40E+05	2.39E+04
F19	Mean	6.96E+05	1.35E+04	9.53E+03	8.77E+03	**4.09E+03**
	Std	4.15E+05	1.23E+04	7.62E+03	9.27E+03	2.08E+03
F20	Mean	2.70E+03	2.77E+03	2.43E+03	2.63E+03	**2.16E+03**
	Std	2.39E+02	1.21E+02	1.24E+02	1.90E+02	6.30E+01
F21	Mean	2.51E+03	2.50E+03	2.36E+03	2.50E+03	**2.34E+03**
	Std	3.03E+01	1.49E+01	1.86E+01	1.79E+01	1.32E+01
F22	Mean	6.72E+03	3.02E+03	2.86E+03	3.06E+03	**2.30E+03**
	Std	1.17E+03	2.28E+03	1.76E+03	2.40E+03	7.77E−01
F23	Mean	3.36E+03	2.85E+03	2.72E+03	2.86E+03	**2.69E+03**
	Std	1.93E+02	1.06E+01	2.79E+01	2.32E+01	2.01E+01
F24	Mean	3.54E+03	3.02E+03	2.89E+03	3.02E+03	**2.87E+03**
	Std	1.52E+02	1.74E+01	1.46E+01	2.20E+01	6.06E+00
F25	Mean	2.96E+03	2.89E+03	2.89E+03	2.89E+03	**2.89E+03**
	Std	2.65E+01	4.44E−01	1.70E−01	1.72E+00	1.60E+00
F26	Mean	8.25E+03	5.68E+03	4.50E+03	5.54E+03	**4.14E+03**
	Std	8.40E+02	1.35E+02	3.00E+02	3.61E+02	1.57E+02
F27	Mean	3.95E+03	3.21E+03	**3.21E+03**	3.22E+03	3.22E+03
	Std	2.00E+02	1.48E+01	1.51E+01	1.67E+01	1.17E+01
F28	Mean	3.45E+03	3.21E+03	**3.20E+03**	3.21E+03	3.21E+03
	Std	7.32E+01	2.42E+01	3.95E+01	3.55E+01	2.21E+01
F29	Mean	4.45E+03	4.14E+03	4.12E+03	4.02E+03	**3.53E+03**
	Std	2.33E+02	1.16E+02	3.01E+02	2.43E+02	1.17E+02
F30	Mean	3.32E+06	1.04E+04	1.25E+04	1.26E+04	**6.88E+03**
	Std	3.57E+06	4.36E+03	8.70E+03	6.07E+03	1.03E+03

Optimal mean values are in bold.

**Table 10:** Comparison of COGBSO with latest competitive algorithms for CEC 2018 test functions with D = 30.

BFs		COGBSO	SSA	MSWOA	MSCA	AOA	QANA
F1	Mean	3.63E+03	6.96E+03	9.50E+03	1.26E+04	4.02E+10	**1.00E+02**
	Std	4.25E+03	6.83E+03	2.90E+03	2.10E+04	6.12E+09	1.02E−14
F3	Mean	3.32E+02	1.46E+04	9.10E+03	6.81E+03	7.38E+04	**3.00E+02**
	Std	6.19E+01	4.54E+03	4.22E+03	7.44E+03	7.93E+03	4.71E−08
F4	Mean	5.06E+02	4.98E+02	**3.34E+02**	4.73E+02	8.61E+03	4.07E+02
	Std	1.17E+01	1.51E+01	4.81E+01	2.81E+01	3.01E+03	2.01E+01
F5	Mean	**5.48E+02**	6.58E+02	6.08E+02	7.01E+02	7.98E+02	6.05E+02
	Std	1.72E+01	7.66E+01	4.93E+01	2.14E+01	3.22E+01	2.75E+01
F6	Mean	**6.00E+02**	6.29E+02	6.01E+02	6.01E+02	6.64E+02	6.00E+02
	Std	5.47E−02	7.56E+00	6.04E−01	5.54E−01	8.18E+00	3.20E−01
F7	Mean	**8.13E+02**	8.82E+02	8.08E+02	1.18E+03	1.31E+03	8.29E+02
	Std	6.60E+01	4.78E+01	2.94E+01	4.60E+01	5.96E+01	2.92E+01
F8	Mean	**8.39E+02**	9.44E+02	1.10E+03	1.06E+03	1.03E+03	8.94E+02
	Std	9.46E+00	3.01E+01	3.69E+01	1.76E+01	2.95E+01	2.75E+01
F9	Mean	**9.04E+02**	3.86E+03	9.93E+02	9.28E+02	5.55E+03	1.18E+03
	Std	5.83E+00	1.69E+03	1.38E+02	1.87E+02	7.95E+02	4.91E+02
F10	Mean	8.07E+03	4.89E+03	7.76E+03	8.19E+03	6.58E+03	**3.38E+03**
	Std	2.93E+02	9.26E+02	8.34E+02	5.47E+02	4.95E+02	6.18E+02
F11	Mean	1.17E+03	1.36E+03	**1.08E+03**	1.37E+03	3.36E+03	1.18E+03
	Std	2.53E+01	9.70E+01	2.05E+01	3.14E+01	1.52E+03	3.48E+03
F12	Mean	3.87E+04	2.76E+07	5.15E+04	1.25E+05	6.77E+09	**3.32E+03**
	Std	1.84E+04	3.58E+07	2.58E+04	3.98E+05	1.75E+09	1.92E+03
F13	Mean	1.04E+04	1.51E+05	1.98E+04	3.03E+04	4.36E+04	**1.38E+03**
	Std	1.65E+04	8.53E+04	1.31E+04	1.68E+04	4.01E+04	4.02E+01
F14	Mean	2.41E+03	2.44E+04	2.39E+04	2.26E+04	4.85E+04	**1.47E+03**
	Std	9.15E+02	2.41E+04	9.40E+02	1.42E+04	3.60E+04	2.14E+01
F15	Mean	4.92E+03	5.94E+04	5.18E+03	6.60E+03	2.47E+04	**1.53E+03**
	Std	3.25E+03	4.67E+04	7.38E+03	6.53E+03	1.10E+04	4.28E+00
F16	Mean	**2.14E+03**	2.75E+03	3.78E+03	3.70E+03	3.65E+03	2.31E+03
	Std	1.63E+02	3.82E+02	3.82E+02	2.73E+02	4.49E+02	2.64E+02
F17	Mean	**1.85E+03**	2.12E+03	2.89E+03	2.60E+03	2.59E+03	1.86E+03
	Std	7.56E+01	1.88E+02	2.42E+02	1.72E+02	3.08E+02	7.25E+01
F18	Mean	7.93E+04	6.13E+05	1.66E+05	3.43E+05	1.03E+06	**5.44E+03**
	Std	2.39E+04	5.17E+05	1.75E+05	1.44E+05	9.97E+05	4.89E+03
F19	Mean	4.09E+03	1.35E+06	4.92E+03	2.28E+04	1.09E+06	**1.97E+03**
	Std	2.08E+03	1.01E+06	2.94E+03	3.11E+04	7.90E+04	1.29E+02
F20	Mean	**2.16E+03**	2.54E+03	2.92E+03	2.68E+03	2.69E+03	2.23E+03
	Std	6.30E+01	2.03E+02	1.67E+02	8.97E+01	1.67E+02	1.14E+02
F21	Mean	2.34E+03	2.43E+03	2.62E+03	2.56E+03	2.57E+03	**2.17E+03**
	Std	1.32E+01	2.18E+01	4.91E+01	1.93E+01	5.09E+01	2.64E+01
F22	Mean	**2.30E+03**	2.30E+03	2.30E+03	2.74E+03	7.84E+03	2.31E+03
	Std	7.77E−01	1.68E−05	6.64E−01	2.55E+02	9.46E+02	3.01E+01
F23	Mean	2.69E+03	2.77E+03	2.62E+03	2.81E+03	3.33E+03	**2.51E+03**
	Std	2.01E+01	3.63E+01	2.06E+01	2.50E+01	1.12E+02	1.66E+01
F24	Mean	2.87E+03	2.94E+03	2.89E+03	2.96E+03	3.60E+03	**2.60E+03**
	Std	6.06E+00	3.33E+01	1.04E+01	1.36E+01	1.53E+02	0.00E+00
F25	Mean	2.89E+03	2.90E+03	2.89E+03	2.97E+03	4.38E+03	**2.70E+03**
	Std	1.60E+00	1.46E+01	1.01E+00	1.11E+00	4.95E+02	0.00E+00
F26	Mean	4.14E+03	4.52E+03	4.23E+03	4.86E+03	9.22E+03	**2.80E+03**
	Std	1.57E+02	1.22E+03	3.77E+02	2.06E+02	8.82E+02	0.00E+00
F27	Mean	3.22E+03	3.24E+03	3.20E+03	3.23E+03	4.09E+03	**2.93E+03**
	Std	1.17E+01	1.43E+01	1.45E+01	1.44E+01	2.38E+02	7.14E+01
F28	Mean	3.21E+03	3.25E+03	3.22E+03	3.27E+03	5.68E+03	**3.00E+03**
	Std	2.21E+01	1.49E+01	2.95E+01	2.21E+01	5.65E+02	0.00E+00
F29	Mean	3.53E+03	3.95E+03	3.50E+03	4.07E+03	5.52E+03	**3.10E+03**
	Std	1.17E+02	2.47E+02	1.07E+02	1.93E+02	6.89E+02	0.00E+00
F30	Mean	6.88E+03	7.03E+06	1.62E+04	8.93E+03	6.01E+07	**3.96E+03**
	Std	1.03E+03	4.30E+06	1.07E+03	3.61E+03	1.76E+08	8.49E+01

Optimal mean values are in bold.

Several conclusions can be drawn by analyzing the optimization accuracy of 15 test functions selected from the CEC2013. First, for the 10-dimensional problems in the comparison of other improved brain storm optimization algorithms, the performance of the COGBSO algorithm is much better than that of the BSO algorithm, and the performance of the relatively improved GBSO, GDBSO, and ADMBSO algorithms are still greatly improved. The COGBSO algorithm has the best performance on all benchmark functions. The main reason is that the COGBSO algorithm combines the advantages of the GDBSO and ADMBSO algorithms to improve the convergence speed of the algorithm. Moreover, the possibility of the algorithm jumping out of the local optimum is significantly increased with the assistance of the chaotic difference step and the opposition-based population strategy, which improvs the optimization accuracy of the algorithm. The COGBSO algorithm still has some advantages compared to other swarm intelligence algorithms. Compared to the SSA algorithm, the COGBSO algorithm performs slightly less on the F9 function and is the absolute leader in performance on the remaining functions. Compared to the MSCA and MSWOA algorithms, the COGBSO algorithm can guarantee the lead in optimization accuracy on most benchmark functions but performs poorly on several test functions.

Second, compared to other improved brain storm optimization algorithms, the performance of the COGBSO algorithm degrades on individual benchmark functions for 20-dimensional problems. On the F12 and F13 benchmark functions, the performance of the COGBSO algorithm is only slightly lower than the GBSO algorithm, higher than the ADMBSO and GDBSO algorithms, and much higher than the BSO algorithm. On the F15 benchmark function, although the performance of the COGBSO algorithm is higher than that of the BSO algorithm, it is lower than the three algorithms of GBSO, GDBSO, and ADMBSO. The global optimal value 0 can be found in 30 experiments, but the number of times is limited. On other benchmark functions, the COGBSO algorithm has the best performance. Compared to other swarm intelligence algorithms, the performance of the COGBSO algorithm is similar to that of the 10-dimensional case. COGBSO still dominates the comparison of the SSA algorithms across the board. Compared to the MSCA and MSWOA algorithms, the COGBSO algorithm can take the lead on eight test functions.

Third, for 30-dimensional problems, compared to other improved brain storm optimization algorithms, the COGBSO algorithm also performs poorly on the three benchmark functions of F12, F13, and F15. However, in general, it still has the best optimization performance and can better optimize the multi-modal function. Compared to other swarm intelligence algorithms, the COGBSO algorithm still takes the lead on eight test functions. The results also prove that no one type of swarm intelligence algorithm can solve all optimization problems perfectly, and all need to choose a more suitable intelligent algorithm according to the problem.

In the experiments on the CEC2018 benchmark test suit, we directly use the complex dimension of 30 dimensions to test the optimization accuracy of the algorithms further, and two new competitive algorithms are added, which are the AOA algorithm and the QANA algorithm, and the following conclusions can be obtained based on the simulation results. The COGBSO algorithm has a significant advantage over the other variants of the BSO algorithm in the CEC2018, leading to optimization accuracy on 22 functions and a tiny gap with the other algorithms on their inferior functions. Compared with other types of competitive, intelligent algorithms, the COGBSO algorithm still has certain advantages; as can be seen from Table 10, the COGBSO algorithm is only weaker than the QANA algorithm in terms of overall performance, but it can still achieve better optimization results on one-third of the functions and is entirely ahead of the other four algorithms. All of these experimental results prove the superiority of the COGBSO algorithm.

**Figure 5:**
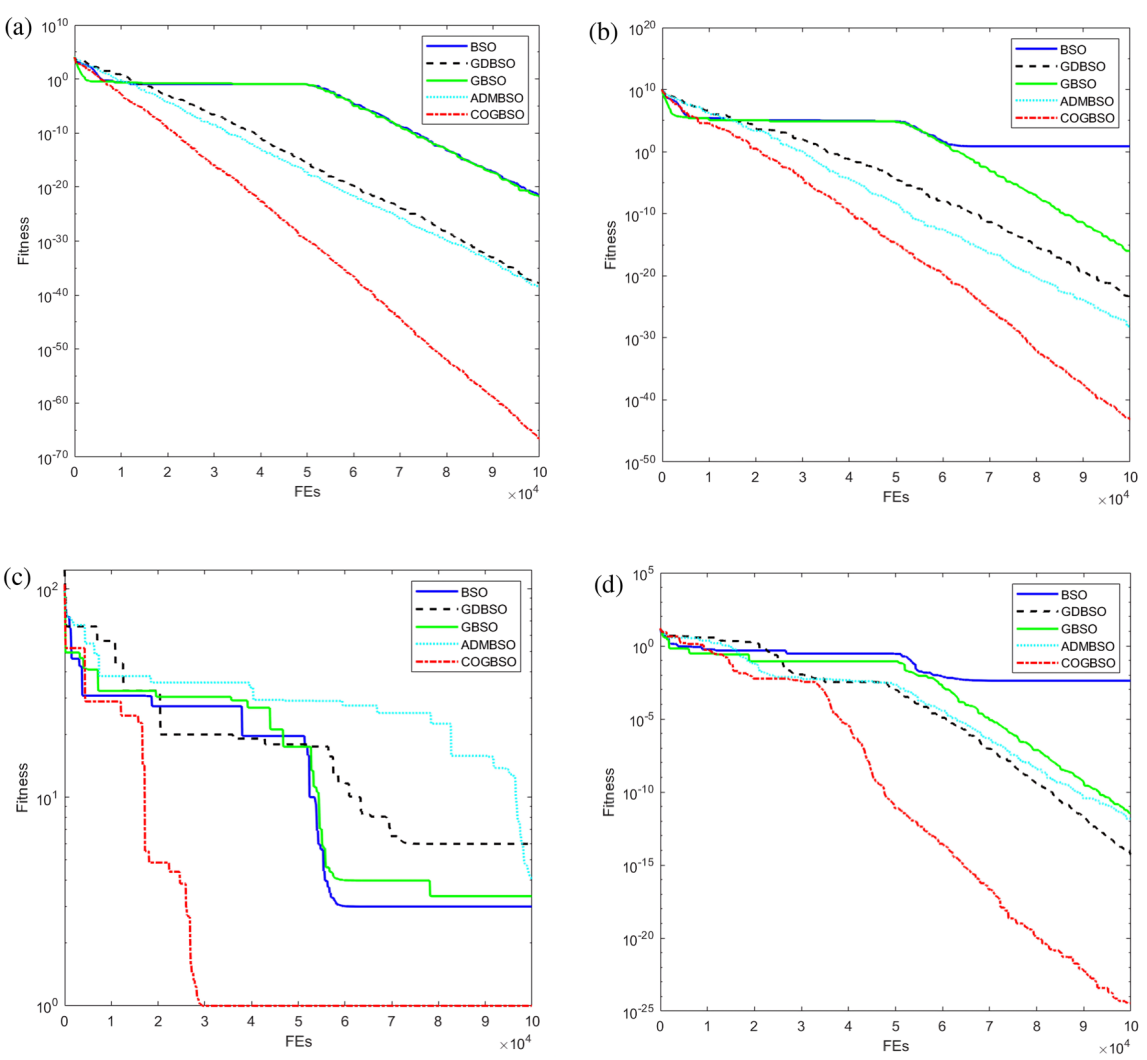
The convergence curves of four typical benchmark functions on 10-D problems.

**Figure 6:**
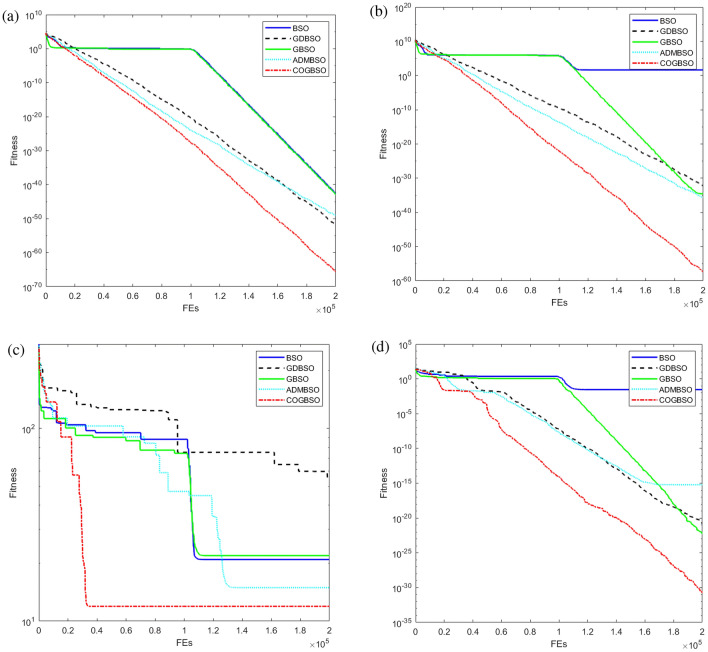
The convergence curves of four typical benchmark functions on 20-D problems.

**Figure 7:**
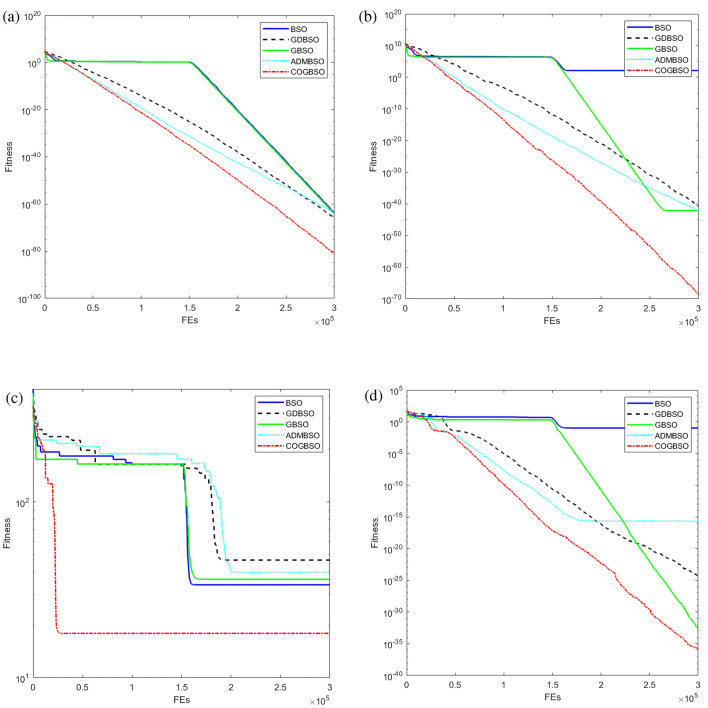
The convergence curves of four typical benchmark functions on 30-D problems.

**Figure 8:**
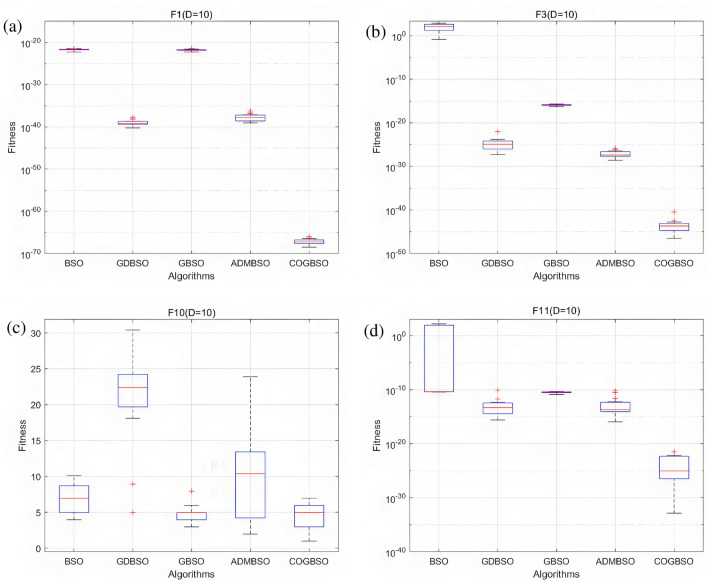
Boxplots between algorithms on F1, F3, F10, F11.

In order to better demonstrate the advantages of the COGBSO algorithm in terms of convergence speed, the convergence curves of the different improved BSO algorithms on four benchmark functions from CEC2013 are shown in Figs. 5, 6, and 7, and the box line diagram situation is shown in Fig. 8. Meanwhile, based on the optimization accuracy results of each algorithm, this paper also provides further evidence of the effectiveness of the COGBSO algorithm through the Friedman test. The results of the non-parametric tests are presented in Tables 11 and 12. In addition, the convergence curve test and Friedman test results are only used to compare the COGBSO algorithm with other improved BSO algorithms to ensure the brevity of the paper. The comparison with other types of swarm intelligence algorithms through the table of optimization accuracy is enough to prove the advantages of the COGBSO algorithm.

**Table 11:** Friedman’s test results for each algorithm.

Algorithm	BSO	GBSO	ADMBSO	GDBSO	COGBSO
Ranking	4.6	3.0667	2.8	2.6	1.9333

**Table 12:** Wilcoxon statistical test results of COGBSO.

Algorithm	p-value	R+	R−
BSO	0.000655	120	0
GBSO	0.000655	120	0
ADMBSO	0.000982	105	0
GDBSO	0.006319	96	9

It can be seen from the figure that COGBSO has better convergence speed and optimization accuracy than other improved BSO algorithms. On the convergence curve of the F10 function, it can be observed that with the assistance of the chaotic difference step search space and the strategy of the opposition-based population, the time for the algorithm to fall into the local optimum is very short. The probability of jumping out of the local optimum is significantly higher than other algorithms. This is because the COGBSO algorithm has a broader search space and a higher probability of generating an optimal solution, which makes it difficult for the algorithm to fall into the trap of local optimum for a long time. The boxplots are only given for the 10-dimensional condition due to space limitations. On the boxplots of the four functions, the COGBSO algorithm has optimal performance on minimum, maximum, and median. The Friedman test was used to obtain the average ranking of each algorithm on all test functions. In the nonparametric statistical test, the lower the ranking, the better performance of the algorithm. Table 11 shows the test results. COGBSO ranked first (1.9333), while GDBSO ranked second (2.6). Moreover, the Wilcoxon statistical test was used to verify the significance of the algorithm, and the results are summarized in Table 12. In comparison with that of COGBSO, the p-value of each algorithm was lower than 0.05, indicating that COGBSO has significant advantages over the other algorithms. In addition, the resistance values R+ and R- reflected the excellent performance of COGBSO. The results once again prove the validity of the COGBSO algorithm.

### Discussion and experimental summaries

**Figure 9:**
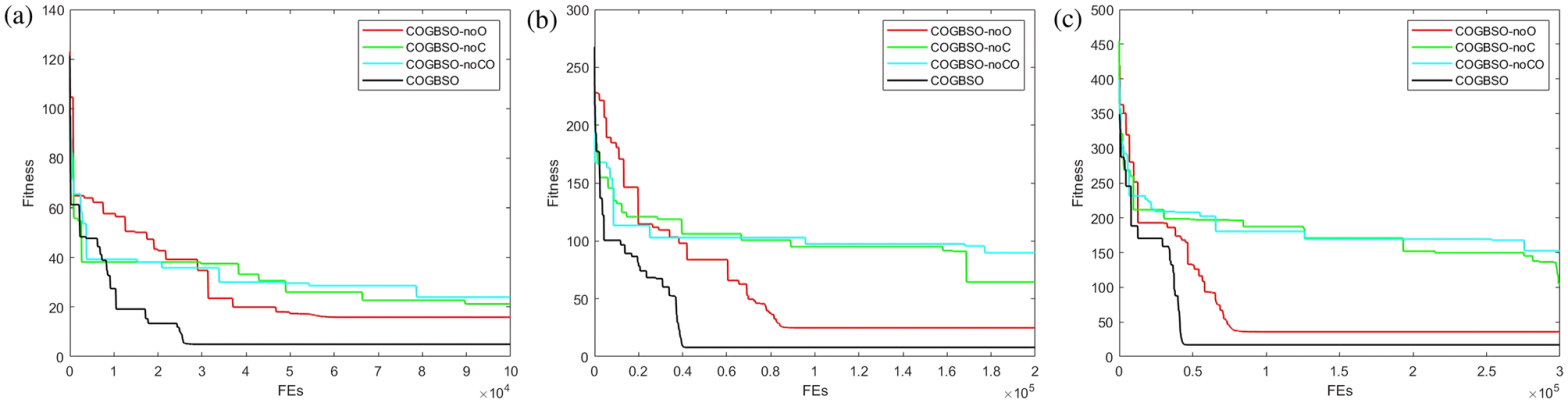
The convergence curves of F10 function based on the idea of ablation experiments.

In order to fully understand the effect of chaotic difference step and opposition-based learning population strategy on jumping out of the local optimal solution, We used the idea of ablation experiments. Additionally, we simulated the optimization of the algorithm in three cases. These three cases are the COGBSO algorithm without the assistance of the chaotic difference step as well as the opposition-based population strategy (COGBSO-noCO), the COGBSO algorithm without the assistance of the chaotic difference step only (COGBSO-noC), and the COGBSO algorithm without the opposition-based population strategy only (COGBSO-noO). Select the typical multi-modal benchmark function F10 (Rastrigin) from CEC2013 for simulation comparison, on the one hand, to ensure the simplicity of the article. On the other hand, the local optimal problem is more likely to appear on the multi-modal function, so using this benchmark function is enough to study the contribution of the chaotic difference step and opposition-based population strategy. The COGBSO-noCO, COGBSO-noC, COGBSO-noO, and COGBSO algorithms are simulated under 10, 20, and 30 dimensions, and the convergence curves are shown in Fig. 9.

The convergence curve shows the superiority of the chaotic difference step and the opposition-based population strategy in jumping out of the local optimal solution. First, the black line in the figure represents the COGBSO algorithm, which has the fastest convergence speed in 10, 20, and 30 dimensions. This result proves the effectiveness of the chaotic difference step with the opposition-based population strategy. Second, it can be observed from the curve that the COGBSO algorithm with chaotic difference step and opposition-based population strategy makes it difficult to fall into the local optimum for a long time before reaching the final solution. Moreover, the COGBSO-noCO algorithm has many cases where it falls into local optimum for a long time and fails to move forward under three dimensions. The COGBSO-noC also has some cases of falling into a local optimum for a long time, but the situation is slightly improved compared to the COGBSO-noCO algorithm. The COGBSO-noO is significantly improved but still not as effective as the COGBSO algorithm, which proves that both the chaotic difference step strategy and the opposition-based population strategy can improve the ability of the algorithm to jump out of the local optimum and the chaotic differential step strategy is more effective, but the two strategies are not conflicting. Thus, they can be applied simultaneously to the algorithm to improve its performance even more. Third, the COGBSO algorithm also has the best performance in optimization accuracy, which is better than other algorithms.

Furthermore, the COGBSO-noO algorithm has a higher optimization accuracy than the COGBSO-noC algorithm, and the COGBSO-noC algorithm outperforms the COGBSO-noCO algorithm in optimization accuracy. This implies that both strategies bolster the algorithm’s search accuracy. This result is well-supported, as the algorithm’s more robust ability to escape from the local optimum leads to better performance during iteration. The results show that the chaotic difference step and the opposition-based population strategy enhance the algorithm’s ability to deal with multi-modal problems, significantly improving the optimization accuracy and the probability of jumping out of the local optimum. Moreover, since the two strategies do not conflict with each other, they can be applied to the improvement of the BSO algorithm at the same time.

Based on all the experimental results in Sect. "Simulation results and analysis" and this section, it can be shown that the COGBSO algorithm has the following advantages. First, the optimization accuracy of the COGBSO algorithm has a significant advantage over other variants of the BSO algorithm. This claim can be corroborated by Tables 3, 4, 5, and 9. The COGBSO algorithm can achieve optimal optimization search results on most functions from the CEC2013 and CEC2018 benchmark test suit. Meanwhile, the nonparametric test results in Tables 11 and 12 and the boxplots in Fig. 8 argue this point. Second, the optimization accuracy of the COGBSO algorithm also has a significant advantage over other types of recent competitive intelligent algorithms. This claim can be corroborated by Tables 6, 7, 8 and 10. These results indicate that the COGBSO algorithm’s overall performance is weaker than that of the QANA algorithm but still manages to gain an advantage over many functions while being significantly better than the other intelligent algorithms. Third, the COGBSO algorithm also converges significantly faster than the other BSO variants. This claim can be corroborated by Figs. 5, 6, and 7. Fourth, the chaotic difference step strategy and the opposition-based population strategy proposed in this paper can significantly improve the probability of the BSO algorithm jumping out of the local optimum. This claim can be corroborated by Fig. 9.

## Conclusion

This paper proposes a global-best brain storm optimization algorithm based on chaotic difference step and opposition-based learning (COGBSO). First, the discussion mechanism and the global-best strategy are combined into the BSO algorithm, improving the algorithm’s convergence speed and optimization accuracy. Second, chaos theory is introduced to design a chaotic difference step to expand the search space of the population, and the opposition-based population is introduced to improve the population density. Both strategies are designed to make it easier for the algorithm to escape from the local optimum when dealing with optimization problems, especially multi-modal function problems. Third, COGBSO and BSO, GBSO, ADMBSO, GDBSO, SSA, MSCA, MSWOA, AOA and QANA algorithms are compared and analyzed in this paper.

The results show that for most benchmark functions in 10, 20, and 30 dimensions, the COGBSO algorithm has the best performance. Experiments proved the superior performance of COGBSO compared to previous BSO improved algorithms and enables the goal of helping the algorithm to jump out of a local optimum quickly. As one of the first algorithms inspired by human’s behaviors, COGBSO demonstrates its great potential in dealing with complex optimization problems. Meanwhile, the chaotic difference step strategy in the COGBSO algorithm and the application of opposition-based learning theory can stimulate us to propose more novel strategies to adapt to more complex problems arriving in quick succession. In the future, the COGBSO will be applied to applications of high dimension and large scale.

Future research directions include proposing an adaptive mechanism to adjust parameters, assisting the algorithm to intelligently choose when to use the chaotic difference step and opposition-based population strategy, reducing the space-time complexity of the algorithm, and applying the COGBSO algorithm to practical engineering optimization problems.

## Data Availability

All data generated or analysed during this study are included in this published article.
